# On the emergence of gravitational-like forces in insect swarms

**DOI:** 10.1098/rsif.2019.0404

**Published:** 2019-11-13

**Authors:** Andy M. Reynolds

**Affiliations:** Rothamsted Research, Harpenden, Hertfordshire AL5 2JQ, UK

**Keywords:** insect swarms, collective behaviours, emergent properties

## Abstract

Okubo (Okubo 1986 *Adv. Biophys.* **22**, 1–94. (doi:10.1016/0065-227X(86)90003-1)) was the first to propose that insect swarms are analogous to self-gravitating systems. In the intervening years, striking similarities between insect swarms and self-gravitating systems have been uncovered. Nonetheless, experimental observations of laboratory swarms provide no conclusive evidence of long-range forces acting between swarming insects. The insects appear somewhat paradoxically to be tightly bound to the swarm while at the same time weakly coupled inside it. Here, I show how resultant centrally attractive gravitational-like forces can emerge from the observed tendency of insects to continually switch between two distinct flight modes: one that consists of low-frequency manoeuvres and one that consists of higher-frequency nearly harmonic oscillations conducted in synchrony with another insect. The emergent dynamics are consistent with ‘adaptive’ gravity models of swarming and with variants of the stochastic models of Okubo and Reynolds for the trajectories of swarming insects: models that are in close accord with a plethora of observations of unperturbed and perturbed laboratory swarms. The results bring about a radical change of perspective as swarm properties can now be attributed to known biological behaviours rather than to elusive physical influences.

## 1. Introduction

Insect swarms do not display the choreographed movements seen in fish schools and bird flocks, but their members do remain in just a small portion of the space available to them [1]. Nonetheless, individuals are behaving collectively rather than interacting independently with visual features on the ground over which swarms tend to form [2]. By drawing an analogy with Newtonian gravitational attraction, Okubo [3] speculated that the interactions between swarming insects produces, on the average, a centrally attractive force that acts on every individual. There is now strong experimental support for such a resultant restoring force in laboratory swarms of *Chironomus riparius* midges [1]. The emergence of this resultant restoring force has been attributed to the insects interacting via long-range gravitational-like forces [4]. This is a tempting possibility because insects are thought to interact acoustically, responding to wing-beat noise whose far-field intensity is expected to decay according to an inverse square law [5]. Gravitational-like interactions would, therefore, arise if one insect reacts to another by accelerating towards the source of the sound with a strength that is proportional to the received sound intensity. Experimental observations of laboratory swarms do, however, provide no conclusive evidence for such long-range forces acting between swarming insects [6]. Instead, insects on average display an approximately equivalent acceleration towards almost any feature of the swarm (nearest neighbour, Voronoi centroid, i.e. towards the emptiest region of space in the insect's vicinity, swarm centre). This suggests that individuals are on average weakly coupled, but also tightly bound to the swarm itself [6].

Here, I show how the resultant forces can emerge from the observed tendency of insects to continually switch between two distinct flight modes: one that is composed of relatively straight to and fro movements and one that consists of higher-frequency oscillations [3,7]. Model formulation is presented in the next section. The new model is shown to be closely related to two successful but seemingly distinct models of insect swarms: the stochastic models of Okubo [3] and Reynolds *et al*. [8] and the ‘adaptive’ gravity models of Gorbonos *et al*. [4]. It is also shown how the new model can account for observations that are beyond the scope of the previous models. This is followed by a Discussion.

## 2. Emergence of gravitational-like interactions at the macroscopic level

### 2.1. Model formulation and properties

Multi-camera stereo-imaging and particle-tracking techniques have provided detailed recordings of the three-dimensional trajectories of *C. riparius* midges within laboratory swarms [1,7]. By performing a time–frequency analysis of these trajectories, Puckett *et al*. [7] showed that the midge flight behaviours can be segmented into two distinct modes: one that is independent and composed of low-frequency manoeuvres and one that consists of higher-frequency nearly harmonic oscillations conducted in synchrony with another midge (velocities tend to be antiparallel). These observations have similitude with the observations of Okubo [3] who remarked that the trajectories of individual midges may be classified into two distinct patterns, one being a ‘loose’ pattern and the other a ‘tight’ pattern. In the loose pattern, an insect exhibits relatively straight to and fro moments that might resemble a pendulum motion. In the tight pattern, an insect exhibits a relatively short, zigzag motion that might resemble a random flight. In practice, most individuals display a pattern that combines these two extremes.

The observations of Puckett *et al*. [7] and Okubo [3] suggest that at long-times (times longer than the velocity autocorrelation timescale), individual flight patterns can effectively be partitioned into episodes of ‘hovering’ and ‘flying’ (diffusing) and that the long-time dynamics can be approximated by a pair of reaction–diffusion equations

2.1 $$\begin{array}{rl} \frac{\partial H}{\partial t} & =-\alpha H+\beta HF\\ \mathrm{and}\;\;\;\frac{\partial F}{\partial t} & =\alpha H-\beta HF+D\frac{\partial^{2}F}{\partial x^{2}}. \end{array}\}$$

Here, *H*(*x*, *t*) and *F*(*x*, *t*) are the densities of hoverers and fliers located at position *x* at time *t*, *α* is the rate at which individuals switch from being hoverers to being fliers (with diffusivity *D*) and *β* sets the rate at which fliers switch to becoming hoverers after interacting with hoverers (an alternative, seemingly very credible model is examined then discounted in the electronic supplementary material, S1). These pairwise interactions (biological behavioural traits) could be mediated either acoustically or visually. When the reaction dynamics are very much faster than the diffusive transport, local equilibrium is established, i.e. *αH* − *βHF* = 0. The stable equilibria are *F* = *α/β*, *H = C* − *F* if the density of individuals (fliers and hoverers) *C* > *α*/*β* and *H* = 0, *F* = *C* if *C* < *α*/*β*.

Adding together the two parts of equation (2.1) under the assumption of local equilibrium gives

2.2 $$\begin{array}{rl} \frac{\partial C}{\partial t} & =D\frac{\partial^{2}F}{\partial x^{2}}\\ & =D\frac{\partial^{2}}{\partial x^{2}}\left(\frac{\alpha }{\beta }\right), \end{array}$$

when *C* > *α*/*β*. This shows that the spatial distribution of all individuals within the swarm is ‘frozen’ in time, since the right-hand side of the diffusion equation, equation (2.2), vanishes. That is, the reaction dynamics exactly cancel out the effects of diffusion, thereby creating a stable swarm. Individual fliers are, nonetheless, diffusing within the confines of the swarm. This is made manifest when equation (2.2) is rewritten as

2.3 $$\begin{array}{rl} \frac{\partial C}{\partial t}=\; & D^{\prime }\frac{\partial }{\partial x}\left[\frac{\partial }{\partial x}\left(\frac{C}{P}\right)\right]\\ & \equiv D^{\prime }\frac{\partial }{\partial x}\;\left[\frac{1}{\sqrt{P}}\frac{\partial }{\partial x}\left(\frac{C}{\sqrt{P}}\right)-\frac{C}{2P}\frac{\partial \ln P}{\partial x}\right]\\ & \equiv D^{\prime }\frac{\partial }{\partial x}\left[\frac{1}{P}\frac{\partial C}{\partial x}-\frac{C}{P}\frac{\partial \ln P}{\partial x}\right], \end{array}$$

where *P* ≡ *C* (is a place holder) and $D^{\prime }=(\alpha /\beta )D$.

The first, second and third forms of equation (2.3) correspond to random walk models

2.4 $$dx=\sqrt{\frac{2D^{\prime }}{P}}d\xi ,$$

2.5 $$dx=\frac{1}{2}\frac{D^{\prime }}{P}\frac{\partial \ln P}{\partial x}dt+\sqrt{\frac{2D^{\prime }}{P}}d\xi$$

2.6 $$\mathrm{and}\;\;\;dx=\frac{D^{\prime }}{P}\frac{\partial \ln P}{\partial x}dt+\sqrt{\frac{2D^{\prime }}{P}}d\xi ,$$

where *x* is the position of an individual at time *t,* d*ξ* are increments of a white noise process with autocorrelation〈d*ξ*(*t*)d*ξ*(*t*^′^)〉 = *δ*(*t* − *t*^′^)dt for noises at time *t* and *t*^′^ and where the amplitudes of the noise terms, $\sqrt{2D^{\prime }/P}$, are evaluated: at the start of each step (the ‘Ito’ interpretation) in equation (2.4); at the mid-point of each step (the ‘Stratonovich’ interpretation) in equation (2.5); and at the end of each step (the ‘Hänggi–Klimontovich’ interpretation) in equation (2.6). This non-uniqueness of the corresponding random walk model arises because the interpretation of the intensity of the coloured driving noise is ambiguous in the long-time limit. The colouring of the driving noise is indicative of there being a feedback from the macroscopic level of description of the swarms in terms of the probability density, *P*, to the microscopic kinematics. An individual's movement is therefore dependent on the global properties of the swarm.

Directly analogous results can be obtained albeit non-analytically using a stochastic model that captures both short- and long-time dynamics (electronic supplementary material, S2).

Equation (2.6) is the long-time limit of a close relative of the stochastic models of Okubo [3] and Reynolds *et al*. [8] for the joint evolution of an insect's position, *x*, and velocity, *u*,

2.7 $$\begin{array}{rl} du & =-\frac{u\sqrt{P^{\prime }}}{T}dt+\frac{\sigma_{u}^{2}}{\sqrt{P^{\prime }}}\frac{\partial \ln P}{\partial x}dt+\sqrt{\frac{2\sigma_{u}^{2}}{T}}d\xi \\ \mathrm{and}\;dx & =udt, \end{array}\}$$

where $P^{\prime }=(\beta /\alpha )P$ and where $\sigma_{u}^{2}$ is a velocity scale rather than a mean-square velocity *per se*. Equation (2.6) is obtained from equation (2.7) as the velocity autocorrelation timescale *T* → 0 with $T\sigma_{u}^{2}\to D$, i.e. *t*/*T* → ∞.

In the models of Okubo [3] and Reynolds *et al*. [8] interactions between the individuals are not explicitly modelled (but they can be as shown in Reynolds [9] and in the electronic supplementary material, S3); rather, their net effect is subsumed into a restoring force term. In the model of Reynolds *et al*. [8], this term is given by $\sigma_{u}^{2}(\partial \ln P/\partial x)$ (i.e. by $-(\sigma_{u}^{2}/\sigma_{x}^{2})x$ for swarms with Gaussian density profiles, as in Okubo's [3] classic model where individuals in the swarm behave on the average as if they are trapped in an elastic potential well). In the new model, equation (2.7), this restoring force is renormalized according to the local density and is given by $\sigma_{u}^{2}/\sqrt{P^{\prime }}\;\partial \ln P/\partial x.$ As a result, the central attraction is relatively low in the core of the swarm where the density is relatively high and relatively high in the outskirts of the swarm where the density is relatively low. This closely mirrors ‘adaptive’ gravity models of insect swarms wherein effective forces (presumed to be acoustic interactions) are renormalized according to the local noise amplitude [4]. In Gorbonos *et al*. [4], this modelling assumption was motivated by the fact ‘that for many animals, the perception of sound is not fixed, but rather adapts to the total sound intensity so that acoustic sensitivity drops when there is strong background noise. This is a common feature of biological sensory organs, preventing damage and their saturation’. It is crucial to bring model predictions in line with observations [4]. By preventing collapse (Jeans instability) it also endows swarms with a natural mechanism for self-stabilization [10]. Here, ‘adaptation’ arises freely and is not imposed on the model. Similarly, the ‘frictional term’, − *u*/*T*, which in the models of Okubo [3] and Reynolds *et al*. [8] causes velocity fluctuations to relax back to their (zero) mean value is here replaced by $-u\sqrt{P^{\prime }}/T$. This modification can be attributed to the interactions between the hoverers and fliers. Note that a similar modification, −*uP*^′^/*T*, is induced when short-range repulsions are incorporated into numerical simulations made with the model of Reynolds *et al*. [8] (electronic supplementary material, S4). The noise term represents fluctuations in the resultant internal force that arise partly because of the limited number of individuals in the swarm and partly because of the non-uniformity in their spatial distribution [3].

For locations in and around the core of swarm, the new model, equation (2.7), reduces (up to multiplicative constants, $\sqrt{P^{\prime }(0)}$) to the model of Reynolds *et al*. [8]. This in turn effectively reduces to Okubo's [3] classic model

2.8 $$\begin{array}{rl} du & =-\frac{u\sqrt{P^{\prime }(0)}}{T}dt-\frac{\sigma_{u}^{2}x}{\sigma_{x}^{2}\sqrt{P^{\prime }(0)}}dt+\sqrt{\frac{2\sigma_{u}^{2}}{T}}d\xi \\ \mathrm{and}\;dx & =udt, \end{array}\}$$

when positions are Gaussian distributed. These models agree well with numerous experimental observations of laboratory swarms [8,9,11,12]. The new model does, however, account for observations that are beyond the reach of previous stochastic models; namely the dependency of effective spring constants on swarm size [4]; the emergence of non-Gaussian velocity statistics [1] and the near constancy of swarm densities [1,2].

### 2.2. Accounting for observations that are beyond the scope of previous models

#### 2.2.1. Effective spring constants

Large laboratory swarms and wild swarms tend to be cylindrical in shape with the central axes orientated vertically (along the *z*-axis) [1,13]. In the cores of swarms with Gaussian density profiles, the restorative force term in equation (2.7) increases linearly with distance from the swarm centre. In these locations, the restorative force can, therefore, be characterized by an effective spring constant, $K=\sigma_{u}^{2}/\sigma_{x}^{2}\sqrt{P^{\prime }(0)}$. For highly cylindrical swarms with *σ_x_* = *σ_y_* ≪ *σ_z_*, equation (2.7) predicts that $K_{x}=K_{y}<K_{z},K_{x}\propto \sigma_{x}^{-1}$ and $K_{z}\propto \sigma_{z}^{-3/2}$. Lower effective spring constants in the *z*-direction are observed in laboratory swarms [1] as are the two different scalings with swarm size [4]. These predictions also closely match the predictions of Gorbonos *et al*.'s [4] adaptive gravity model. Nonetheless, if individuals were interacting with one another via long-range gravitational-like forces then all swarms would be spherical in shape, or nearly so. If, on the other hand, swarms are bound together by the interactions between hoverers and fliers, then any shape swarm is possible (stable) in principle (equation (2.2)). Swarm shapes may, however, be constrained by the nucleation process (electronic supplementary material, S5). In contrast with the new model, equation (2.7), Okubo's [3] stochastic model predicts contrary to experiment a single scaling with swarm size, namely $\;K_{x}=K_{y}\propto \sigma_{x}^{-2}$ and $K_{z}\propto \sigma_{z}^{-2}$.

#### 2.2.2. Accounting for the emergence of non-Gaussian velocity statistics

**Figure 1.**
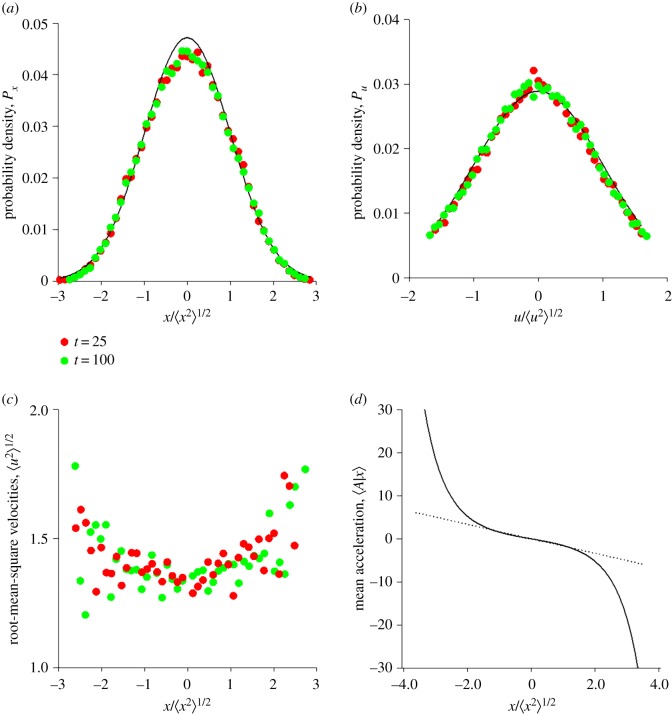
(*a*,*b*) Swarms are predicted to have stationary position and velocity statistics. (*c*) Root-mean-square velocities are predicted to be approximately homogeneous within the swarm's core. (*d*) Individuals are predicted to be effectively bound to the centre of the swarm by a force (mean acceleration 〈*A*|*x*〉) which in the core of the swarm grows linearly with distance from the swarm centre. Predictions are shown at times *t* = 25 (red circles) and *t* = 100 (green circles) together with the best-fit Gaussian distributions (solid-lines). Predictions are shown for equation (2.7) with $P(x)=1/\sqrt{2\pi }\sigma_{x}\exp(-(x^{2}/2\sigma_{x}^{2})),\sigma_{x}=1,\sigma_{u}=1,\alpha =1$ and *β* = 1. (Online version in colour.)

**Figure 2.**
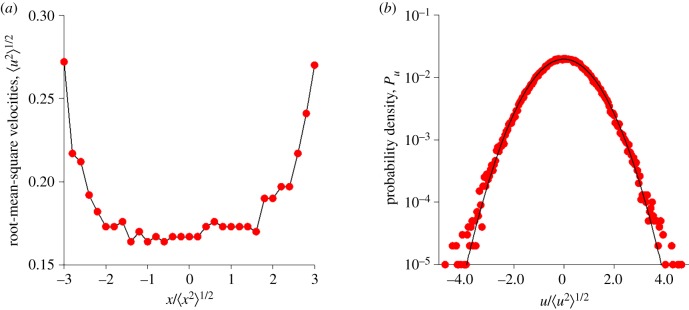
Hallmarks of model predictions in laboratory swarms. (*a*) Root-mean-square velocities profiles are consistent with theoretical expectations (figure 1*c*). (*b*) In accordance with observations [1] velocity distributions of large swarms are predicted to have Gaussian cores and exponential tails. Data (red circles) are taken from [14]. All 17 dusk-time swarms. The line is added to guide the eye. Predictions (red circles) were obtained using the new stochastic model, equation (2.7), with $P(x)=1/\sqrt{2\pi }\sigma_{x}\exp(-(x^{2}/2\sigma_{x}^{2})),\sigma_{x}=5,\sigma_{u}=1,\alpha =1$ and *β* = 1 arb. units. Shown for comparison is a Gaussian distribution with equivalent mean and variance (solid-line). (Online version in colour.)

For locations in the outskirts of the swarm the new model, equation (2.7), departs from the models of Okubo [3] and Reynolds *et al*. [8]. It predicts that velocity statistics are heterogeneous rather than homogeneous (position-independent) (figure 1*a–c*) and it predicts that mean accelerations grow nonlinearly rather than linearly with distance from the swarm centre (figure 1*c*). The former prediction is supported by observations (figure 2*a*). In accordance with model expectations, the velocity-variance profile is concave. This is consistent with the velocities of solitary insects being generally higher than the velocities of insects within swarms [2]. It is also consistent with velocity (and speed) distributions having nearly exponential tails that develop with increasing swarm size [1] (figure 2*b*, electronic supplementary material, S6). The latter prediction is consistent with simulation data produced by Gorbonos *et al*.'s [4] adaptive gravity model and more tentatively with experimental observations [3]. Note that in the stochastic models of Reynolds *et al*. [8], velocity statistics are a model input and not a model prediction.

#### 2.2.3. Near constant densities

The density of insects within laboratory swarms of midges is approximately constant [1,2]. This is different from what has been observed for bird flocks where the number density can fluctuate hugely from flock to flock [15]. In contrast with previous models [3,4,8], this constancy is predicted by the new model. The total number of fliers within a swarm of size *R_s_* is predicted to be *N_F_* = (*α*/*β*)*R_s_* (see text relating to equation (2.1))*.* Therefore, the total number of individuals within a swarm *N* ∝ *R_s_* since *N_F_* ≫ *N_H_* [7]. This constancy although accidental may be significant because it implies that the continual flow of individuals into and out of a swarm [1,14,16] drives changes in swarm morphology. Somewhat counterintuitively such fluctuations are predicted to endow swarms with stabilizing macroscopic mechanical properties similar to solids, including a finite Young's modulus and yield strength [11], properties which have been observed in the laboratory [16]. The fluctuations also have the potential to change fundamentally the characteristics of individual flight patterns. Reynolds and Ouellette [17] showed that the centre of mass fluctuations allow for the emergence of Lévy flight patterns which have subsequently been linked to population maintenance in energetic environments [18].

### 2.3. Alternative models

Equation (2.6) can be recovered from other variants of the models of Okubo [3] and Reynolds *et al*. [8]. It can, for example, be recovered from

2.9 $$\begin{array}{rl} du & =-\frac{uP}{T^{\prime }}dt+\sigma_{u}^{2}\frac{\partial \ln P}{\partial x}dt+\sqrt{\frac{2\sigma_{u}^{2}P}{T^{\prime }}}d\xi \\ \mathrm{and}\;dx & =udt, \end{array}\}$$

where $T^{\prime }=(\alpha /\beta )T$. Despite its appeal, this and other such variants are incompatible with the observed near homogeneity of velocity statistics within the core of a swarm [8] and with the near constancy of the Lagrangian velocity structure function, 〈Δ*u*^2^〉 (K van der Vaart 2019, private communication).

### 2.4. Accounting for speed-dependent forces

**Figure 3.**
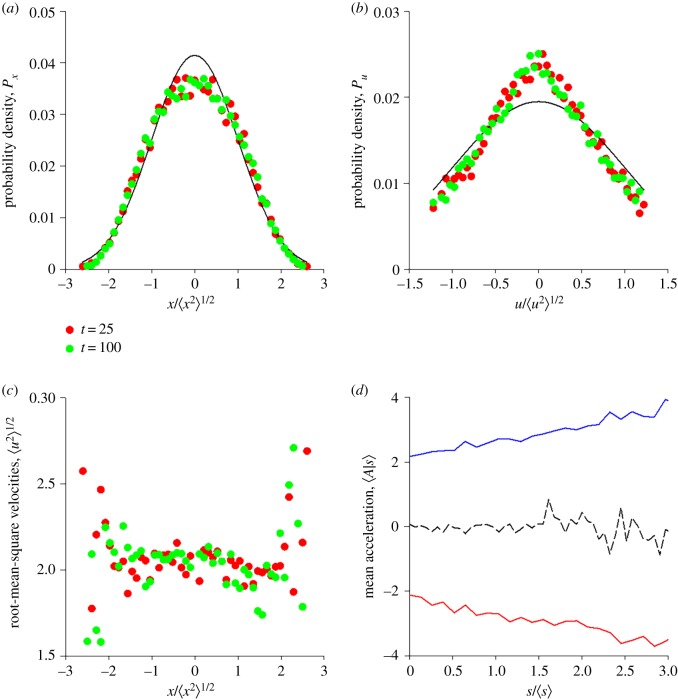
(*a*,*b*) Swarms are predicted to have stationary position and velocity statistics when interactions are speed dependent. (*c*) Root-mean-square velocities are predicted to be approximately homogeneous within the swarm's core. (*d*) Individuals are predicted to be effectively bound to the centre of the swarm by a force (mean acceleration 〈*A*|*s*〉) that increases with an individual's flight speed in accordance with observations [8] (red line shows data for right side only, blue line show data for left side only; and dashed line shows data for both sides which is close to zero, as required by symmetry). Predictions are shown at times *t* = 25 (red circles) and *t* = 100 (green circles) together with best fit Gaussian distributions (solid-lines). Predictions are shown for equation (2.7) with $P(x)=1/\sqrt{2\pi }\sigma_{x}\exp(-(x^{2}/2\sigma_{x}^{2})),\sigma_{x}=1,\sigma_{u}=1,\alpha =1$ and $\beta =\sigma_{u}^{2}/(\sigma_{u}+|u|)$ arb. units. (Online version in colour.)

The foregoing analysis does not directly encompass one of the most intriguing observations: namely the observed speed-dependency of the resultant attractive force [8]. Nonetheless, such a dependency is not unexpected given that the resultant force is here attributed to the interaction between ‘hoverers’ and ‘fliers’ which is itself predicated on movement detection. It is, therefore, seemingly natural to suppose that the rate parameter, *β*, (which governs the interactions between ‘hoverers’ and ‘fliers’ and which has the dimensions of velocity) is, in fact, speed dependent. The simple parameterization $\beta =\sigma_{u}^{2}/(\sigma_{u}+|u|)$ results in stable swarms which in accordance with observations [1,8]: have Gaussian density profiles (figure 3*a*); velocity distributions with Gaussian cores and exponential tails (figure 3*b*); nearly homogeneous velocity statistics (figure 3*c*); and speed-dependent resultant forces which increase monotonically with an individual's speed (figure 3*d*). Comparable predictions are obtained with other simple, biologically plausible, parameterizations of *β* that decrease monotonically with increasing speed.

## 3. Discussion

Stochastic and mechanistic models of insect swarms that draw inspiration from self-gravitating systems are gaining traction because they agree well with experimental observations [3,4,8,9,12,19]. The stochastic model of Reynolds *et al*. [8] is, for example, in close quantitative agreement with data from high-precision, carefully controlled laboratory experiments [1,12,14,16,20,21]. It predicts correctly that swarms consist of a core ‘condensed’ phase surrounded by a dilute ‘vapour’ phase [9] and it predicts correctly that swarms possess emergent continuum mechanical properties, displaying a collective viscoelastic response to applied oscillatory visual stimuli [12]. Moreover, mathematical analysis of the model explains why swarms of flying insects have macroscopic mechanical properties similar to solids, including a finite Young's modulus and yield strength [11]. The mathematical analysis also revealed why in contrast with laboratory insect swarms, wild insect swarms display significant coordinated behaviour [19]. This showed how the presence of a fluctuating environment drives the formation of a transient, local order (synchronized subgroups), and that this local order pushes the swarm as a whole into a new state that is robust to environmental perturbations. At same the time, striking similarities between insect swarms and self-gravitating systems are being uncovered ([4,10,19]; electronic supplementary material, S7–S11). Nonetheless, this success need not be attributed to insects interacting with one another via gravitational-like forces which would be an over interpretation of experimental observations [6]. Here, I showed how resultant gravitational-like forces can emerge from the observed tendency of insects to continually switch between non-diffusive and diffusive flight modes. In other words, the sporadic formulation of bound pairs was shown to be sufficient to bind the swarm together. The emergent resultant gravitational-like forces were found to be consistent with Gorbonos *et al*.'s [4] adaptive gravity model rather than with Newtonian gravity. That is, the resultant central attraction was predicted to be relatively low in the core of the swarm where the density is relatively high and relatively high in the outskirts of the swarm where the density is relatively low. The emergent dynamics were also found to be consistent with variants of the stochastic models of Okubo [3] and Reynolds *et al*. [8]; models that faithfully reproduce many observations made in the laboratory [3,8,9,11,12]. These models can, therefore, be reinterpreted in a radically new way that is biological rather than physical and in a way that this is rooted firmly in observations [3,7] rather than challenged by them [6]. The new analysis suggests that despite their success the models of Okubo [3] and Reynolds *et al*. [8] are effective (phenomenology) models. It also suggests that the success of Gorbonos *et al*.'s [4] adaptive gravity model can be attributed to the fact that it will necessarily predict the emergence of resultant gravitational-like forces and not because it is founded on a realistic representation of the way in which insects interact with one another. The new analysis thereby provides a bridge between the stochastic models of Okubo [3] and Reynolds *et al*. [8], and the manifestly gravitational model of Gorbonos *et al*. [4] by showing how both kinds of model encapsulate similar dynamics and how both can be freed from their original formulations. Moreover, the new stochastic models were shown to predict correctly features of insect swarms (e.g. anisotropic scaling of effective spring constants, the constancy of density) that are beyond the scope of the models of Okubo [3] and Reynolds *et al*. [8] but within reach of adaptive gravity models [4]. Conversely, it reconciles the notion of adaptive gravity with the existence of highly cylindrical wild swarms [13] and with speed-dependent accelerations [8]. The new analysis also shows how the behaviour of swarms studied in quiescent laboratories can be reconciled with the behaviours of wild swarms which must contend with environmental disturbances. In contrast with laboratory swarms, wild swarms form transient synchronized subgroups that push the swarms into the new state that is robust to environmental perturbations [19]. This behaviour (i.e. this strengthening of the effective gravity) may now be seen as an extension of the behaviour (formulation of transient bound pairs) that underlies the emergence of effective gravity itself.

## Supplementary Material

Supplementary Material

## Data accessibility

Computer codes can be obtained from the author.
