# Supplementary material for: On the emergence of gravitational-like forces in insect swarms
Source: J R Soc Interface. 2019 Nov 13;16(160):20190404. doi: 10.1098/rsif.2019.0404 (PMC6893498; doi:10.1098/rsif.2019.0404)
Supplement: Supplementary Material [file rsif20190404supp1.docx]

**Supplementary Material**

**S1. A putative fission-fusion model**

Here an alternative, seemingly very credible model for stable insect swarms is examined then discounted. It may, however, find application in non-stable situations [Supplementary Material S9].

Puckett et al. [2015] showed that midges continually switch between distinct flight modes: one that is independent and composed of low-frequency maneuvers, and one that consists of higher-frequency nearly harmonic oscillations conducted in synchrony with another midge. This behaviour could potentially be regarded as a fusion-fusion process involving singletons, $N_{1}$, and pairs, $N_{2}$,$N_{1}+N_{1}\rightleftarrows_{\beta}^{\alpha} N_{2}$. In this case the long-time dynamics can be described by a pair of reaction-diffusion equations

$\begin{matrix} \frac{\partial N_{1}}{\partial t}=-\alpha N_{1}^{2}+2\beta N_{2}+D_{1}\frac{\partial^{2}N_{1}}{\partial x^{2}} \\ \frac{\partial N_{2}}{\partial t}=\frac{1}{2}\alpha N_{1}^{2}-\beta N_{2}+D_{2}\frac{\partial^{2}N_{2}}{\partial x^{2}} \end{matrix}$ (S1)

where the diffusivity, $D_{2}$, of the pairs is negligible compared with the diffusivity, $D_{1}$, of the singletons. When the reaction dynamics are very much faster than the diffusive transport, local equilibrium is established, i.e., $\alpha N_{1}^{2}-2\beta N_{2}\simeq0$, and Eqn. S1 reduces to two diffusion equations which when added together give

$\frac{\partial N_{1}}{\partial t}=D_{1}\frac{\partial^{2}N}{\partial x^{2}}$ (S2)

where$N=N_{1}+N_{2}$. In the two limits, $N_{1}>>N_{2}$and $N_{2}>>N_{1}$, Eqn. S2 becomes

$\begin{matrix} \frac{\partial N}{\partial t}=D_{1}\frac{\partial^{2}N}{\partial x^{2}} \\ \frac{\partial N}{\partial t}=\sqrt{\frac{2\beta}{\alpha}}D_{2}\frac{\partial^{2}\sqrt{N}}{\partial x^{2}} \end{matrix}$ (S3)

The first case which is consistent with the observed predominance of singletons [Puckett et al. 2015] corresponds to normal diffusion, the latter case corresponds to superdiffusion [Reynolds and Geritz 2015, Supplementary Material S9]. Neither is consistent with the formation of a stationary swarm (in which diffusion is ‘frozen’). Nor are they consistent with the emergence of a resultant centrally-attractive force.

**S2. Gravitational-like forces emerge from intermittent elastic coupling between pairs of individuals: capturing both short- and long-time dynamics**

Here it is shown how gravitational-like forces can emerge from intermittent elastic coupling between pairs of insects; in a way that captures both short- and long-time dynamics. The emergence of resultant restorative forces from intermittent coupling may explain why insects appear to be tightly bound to the swarm while at the same time weakly coupled inside it [Puckett et al. 2014]. The underlying dynamics are here modelled by

$\begin{matrix} d\mathbf{u}_{i}=-\mathbf{u}_{i}dt+k_{ij}\left(t\right)\left(\mathbf{x}_{j}-\mathbf{x}_{i}\right)dt+\sqrt{2}d\mathbf{\xi}_{i} \\ d\mathbf{x}_{i}=\mathbf{u}_{i}dt \end{matrix}$ (S4)

where $\mathbf{x}_{i}$and$\mathbf{u}_{i}$denote the 3-dimensional position and velocity of the i^th^ individual in the swarm at time *t* , $\mathbf{x}_{j}$is the 3-dimensional position of the j^th^ individual,$k_{ij}\left(t\right)$ is a coupling constant, and $d\xi$ are increments of a white noise process with autocorrelation$\left\langle d\xi\left(t\right)d\xi\left(t^{/}\right)\right\rangle=\delta\left(t-t^{/}\right)dt$. In the absence of time-dependent coupling, individual motions are described by an Ornstein-Uhlenbeck process, i.e., by a first-order autoregressive process. In this case velocities are Gaussian distributed with mean zero and unit variance (a.u.). And movements are ballistic over time intervals less than the velocity autocorrelation timescale *T=1* (a.u.) and are diffusive over long times. The coupling is consistent with the observations of Puckett et al. [2015]. Puckett et al. [2015] reported that in laboratory swarms of the midge *Chironomus riparius* individual insects tend to continually switch between two distinct flight modes: one that consists of lower frequency maneuvers and one that consists of higher-frequency nearly harmonic oscillations conducted in synchrony with another insect. The coupling arrests relative dispersion and consequently results in the formation of a statistically stationary swarm (Fig. S1a). Coupling also leads to the emergence of gravitational-like forces, i.e., to resultant forces (mean accelerations) that bind individuals to the swarm centre and which increase linearly with distance from the swarm centre (Fig. S1b). This is consistent with observations [Okubo 1986, Kelley and Ouellette 2012] and is indicative of a self-gravitating system [Okubo 1986]. Linear resultant restorative forces are also obtained when the coupling between individuals is nonlinear, e.g., when the linear coupling in the model, Eq. S4., is replaced either by $k_{ij}\left(t\right)\left(\mathbf{x}_{j}-\mathbf{x}_{i}\right)\left|\mathbf{x}_{j}-\mathbf{x}_{i}\right|$ or by $k_{ij}\left(t\right)\mathrm{sgn}\left(\mathbf{x}_{j}-\mathbf{x}_{i}\right)\left|\mathbf{x}_{j}-\mathbf{x}_{i}\right|^{1/2}$. Moreover, as observed [Reynolds et al. 2017], emergent resultant forces are speed dependent (Fig. S1c). The modelling may also account for the occurrence of ellipsoid-shaped swarms [Kelley and Ouellette 2013] which is not possible in truly self-gravitating systems; but can arise here if the coupling strength and/or the likelihood of coupling is dependent on direction.


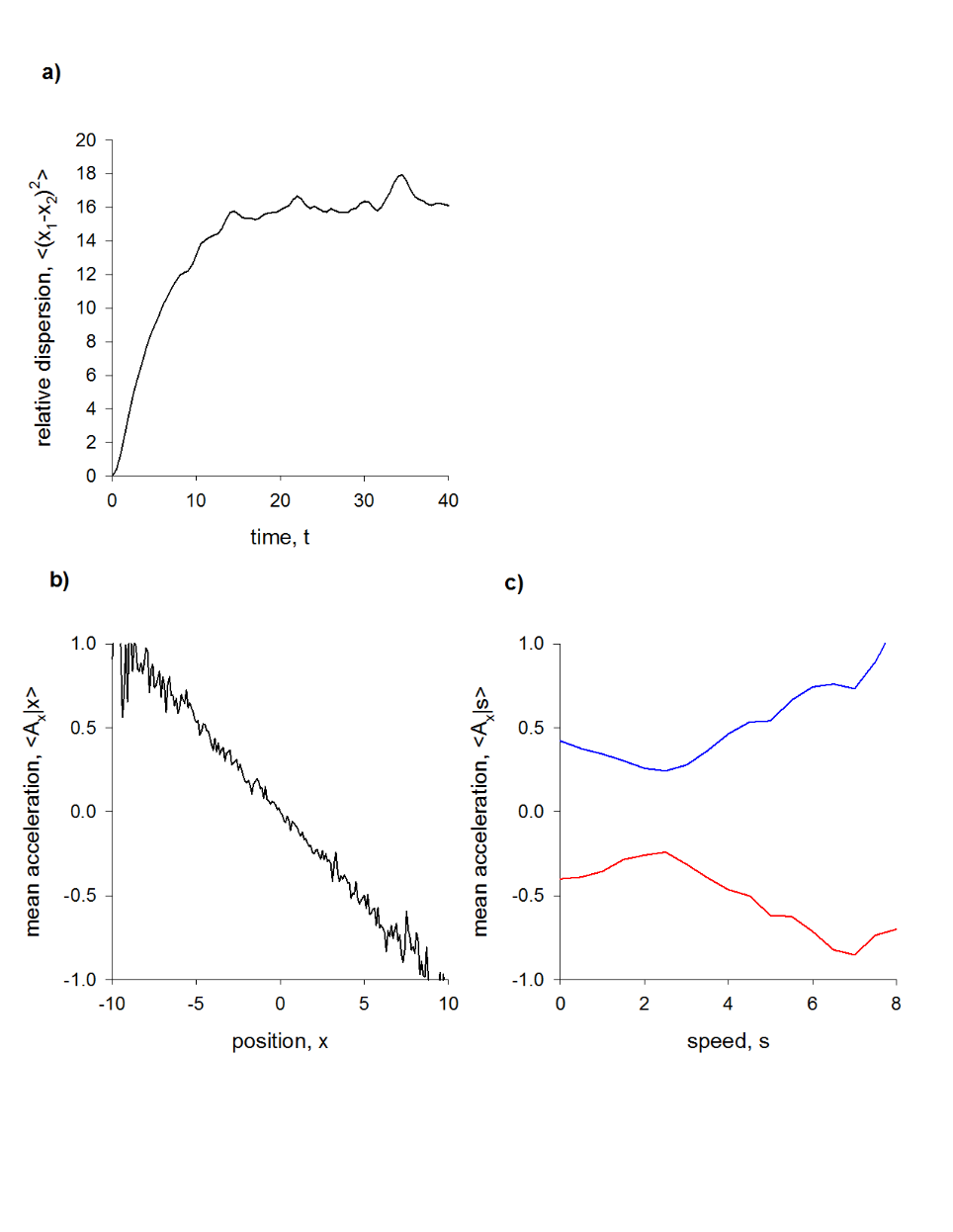


**Figure S1)** **Model predictions for a swarm containing 10 intermittently-coupled individuals. a)** **Coupling arrests relative dispersion, resulting in the formation of a statistically-stationary swarm. b) Coupling leads to the emergence of gravitational-like forces, i.e., to resultant forces (here the x-component of the mean acceleration is shown) that bind individuals to the swarm centre and which increase linearly with distance from the swarm centre. c) The emergent forces are speed dependent.** At any time just 1 randomly chosen pair of individuals are coupled (*k_ij_=1* a.u*.*). All other individuals are uncoupled (*k_ij_=0*). The coupling persists for a time $\Delta t=0.5$ a.u. Thereafter a new randomly chosen pair of individuals are coupled. And so on. Simulation data are shown for a single component of acceleration. The blue line shows data only for the left hemisphere, and the red line only for the right hemisphere.

**S3. Incorporating interactions into the model of Okubo model**

Okubo [1986] did not explicitly model interactions between individuals; rather their net effect was subsumed into a resultant force term. By analogy with Newtonian gravitational attraction, Okubo [1986] speculated that the resultant internal attraction produces, on average, a centrally attractive force that acts on each individual. Here I posit a slightly more elaborate model that takes partial account of interactions between individuals. In contrast with Okubo’s [1986] model, this new model predicts correctly that swarms effectively ‘solidify’ (stabilize, becoming more robust to environment perturbations) rather than ‘melt’ (destabilize) as they grow in size [Ni and Ouellette 2016]. The new model is also shown to encapsulate observed features of swarming that are beyond the scope of Okubo’s [1986] model. Here I assume that individuals are attached to the *instantaneous* centre-of-mass of their companions rather than to the centre-of-mass of the whole swarm (because individuals are not attracted to themselves). That is, I assume that the likelihood of finding individual “*1”* at some position$x_{1}$ in a swarm with *N* individuals is

$p\left(x_{1}\left|x_{2},x_{3},...,x_{N}\right.\right)=\frac{1}{\sqrt{2\pi}\sigma_{x}}\exp\left(-\frac{\left(x_{1}-\bar{x}_{1}\right)^{2}}{2\sigma_{x}^{2}}\right)$ (S5)

where $\overline{x}_{_{1}}=\frac{1}{N-1}\sum_{i=2}^{N} x_{i}$. Short range repulsion, which is observed [Ni and Ouellette 2015], is not accounted for here. All other individuals, “2”, “3”,…”N”, are distributed in a directly analogous way so that

$P\left(x_{1},x_{2},x_{3},...,x_{N}\right)=p(x_{1}|x_{2},x_{3},...,x_{N})p(x_{2}|x_{2},x_{3},...,x_{N})...$ (S6)

Integrating, Eqn.S6, over all but one individual shows that the swarm is not localized in space but is instead freely roaming. The joint distribution, Eqn. S6, is maximal at $x_{1}=x_{2}=...x_{N}$.

Weak localization does, however, arise if each individual is attracted to both its companions and to a swarm marker (located at *x=0*) so that $\overline{x}_{_{1}}=\frac{1}{N}\sum_{i=2}^{N} x_{i}$etc. In accordance with observations [Puckett and Ouellette 2014], the influence of the swarm marker is predicted to wane as the swarm grows in size. Stronger localization can be modelled in an analogous way. In the case of weak localization, the joint distribution, Eqn. S6, is maximal at the location of the swarm marker$x_{1}=x_{2}=...x_{N}=0$and unconditional distributions of position are Gaussian distributed with mean zero and variance, $\sigma_{x}^{2}$, in broad agreement with observations [Kelley and Ouellette 2013]. Reynolds and Ouellette [2016] showed how to construct trajectory simulation models that are exactly compatible with these position statistics and with prescribed velocity statistics. If velocities are Gaussian distributed, then the resultant force term in this model is given by

$F_{i}=-\left(1+N^{-1}+N^{-2}\right)\frac{\sigma_{u}^{2}}{\sigma_{x}^{2}}\left(x_{i}-\left(N^{-1}+2N^{-2}\right)\sum_{\begin{matrix} j=1 \\ j\neq i \end{matrix}}^{N} x_{j}\right)$ (S7)

As $N\to\infty$ $F_{i}=-\frac{\sigma_{u}^{2}}{\sigma_{x}^{2}}x_{i}$, which is the form posited by Okubo [1987] and the one observed in laboratory swarms [Okubo 1987, Kelley and Ouellette 2013]. But in contrast with Okubo’s [1986] model, individual positions are correlated as $\left\langle x_{i}x_{j}\right\rangle=\frac{N+2}{N+3}\left\langle x_{i}^{2}\right\rangle$(where $i\neq j$). The new model therefore predicts that the mean-square centre-of-mass position is given by $\left\langle x_{c}^{2}\right\rangle=\frac{1}{N^{2}}\sum_{i,j=1}^{N} \left\langle x_{i}x_{j}\right\rangle\approx\sigma_{x}^{2}$whereas Okubo’s [1987] model predicts that $\left\langle x_{c}^{2}\right\rangle=\sigma_{x}^{2}/N$ That is, the new model predicts $\left\langle x_{c}^{2}\right\rangle\propto N^{2/3}$whereas Okubo’s [1987] model predicts that$\left\langle x_{c}^{2}\right\rangle\propto N^{-1/3}$ because the number density of insects in a swarm is approximately constant, i.e., $\sigma_{x}\propto N^{1/3}$ [Kelly and Ouellette 2013]. This may be a significant difference because Reynolds [2019] showed that the observed solid-like properties of swarms (a finite Young’s modulus and yield stress) [Ni and Ouellette 2016] can, somewhat counter-intuitively, be attributed to be attributed to centre-of-mass movements. The new model therefore predicts that swarms effectively ‘solidify’ as they increase in size whereas Okubo’s [1986] model predicts that they effectively melt (lose their solid-like properties). This distinction is not without biological significance because male midges swarm to provide a mating target for females, making stationarity (tensile strength) desirable. This new model indicates that swarm size is not constrained by the need for stationarity, re-enforcing biological explanations for swarm size distributions [Neems et al. 1992].

The new model may also account for the observed statistical clustering of individuals within laboratory swarms [Kelley and Ouellette 2013]. The mean spacing between non-interacting individuals in a swarm with a Gaussian density profile is predicted to be $\left\langle\left(x_{i}-y_{i}\right)^{2}\right\rangle=\sigma_{x}^{2}$ whereas for *N* interacting individuals in spatially-localized Gaussian swarms, $\left\langle\left(x_{i}-y_{i}\right)^{2}\right\rangle=\frac{2}{N+3}\sigma_{x}^{2}$ (where $i\neq j$). Interacting insects are therefore predicted on average to be closer together than are non-interacting insects. The new model also predicts, in accordance with observations [Kelley and Ouellette 2013], that root-mean-square nearest-neighbour distances decrease as swarms grow more populous.

**S4. Interactions can result in density-dependent memory terms**

Here it shown that density-dependency of memory terms in stochastic models, e.g., Eqn. 7, can be attributed to interactions between individuals. Swarms consisting of 10 individuals were simulated using the model of Okubo [1986]. Apart from short range repulsion these individuals were non-interacting, Short range repulsion [Ni and Ouellette 2015] was implemented by reflecting the velocities of individuals that get sufficiently close to another. The memory term in Okubo’s [1986] model can be recovered from simulated trajectories by averaging over velocity increments conditioned on both velocity and absolute distance from the swarm centre. In the presence of short range repulsion, this procedure reveals that the effective memory term is dependent on position (Fig. S2a). This is simply a consequence of repulsion (reflection) curtailing the velocity autocorrelation and the fact that this occurs most frequently in the core of the swarm. Rescaling reveals that the effective memory term is proportional to the swarm density profile (Fig. S2b).


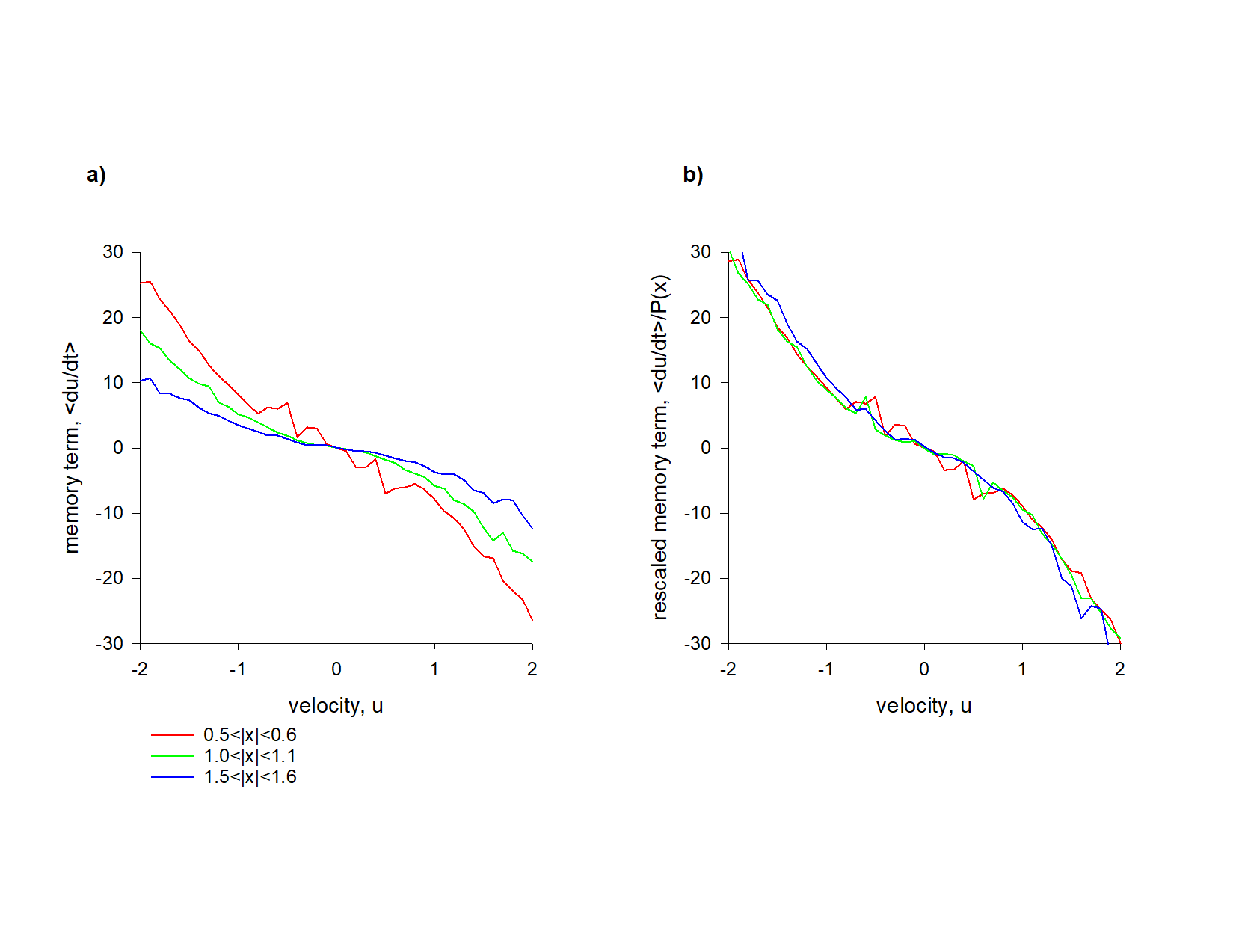


**Figure S2) Short range repulsion results in density-dependent memory terms. a) Averaged velocity-increments conditioned on velocity, *u*, and on position, *x*, reveals the density dependency of the effective memory term. b) Conditional averages collapse when rescaled by the density profile,$P\left(x\right)$, showing that the effective memory is proportional to $P\left(x\right)$.** Predictions were obtained using Okubo’s [1986] model with $\sigma_{x}=1, \sigma_{u}=1$and *T=1* a.u. to track 10 individuals simultaneously. Short range repulsion was implemented at a range 0.01 a.u.

**S5. Speculative ideas about nucleation**

The new model, Eqn. 7, predicts that any swarm shape is stable. If correct then it follows that swarm shapes are determined by the nucleation process and not by the swarm dynamics that *maintain* cohesiveness. Larger swarms have density profiles that are closely Gaussian [Kelley and Ouellette 2013]. Smaller swarms have density profiles with Gaussian cores and heavier tails [van der Vaart, Private Communication]. Here it is shown how these observations can be attributed to individual attraction to a visual feature (a ‘swarm marker’) on the ground over which swarms tend to form. This swarm nucleation process can be modelled by simple variants of Okubo’s [1986] classic model in which the term describing the resultant internal attraction to the swarm centre, $\left\langle A\left|x\right.\right\rangle=-\frac{\sigma_{u}^{2}}{\sigma_{x}^{2}}x$, is replaced by a term describing attraction to the swarm marker. Here I posit that the attraction to the swarm marker is limited in range and described by

$\left\langle A\left|x\right.\right\rangle=-\frac{\sigma_{u}^{2}}{\sigma_{x}^{2}}\frac{x}{1+\frac{x^{2}}{\Lambda^{2}}}$ (S8)

The precise form of the attraction is not important as comparable results are obtained when the range of influence of the swarm maker, *Λ*, is limited in other ways. The associated density profile, *ρ*, is determined by $\frac{\sigma_{u}^{2}}{\rho}\frac{d\rho}{dx}=\left\langle A\left|x\right.\right\rangle$ [Reynolds and Ouellette 2016] and so given by

$\rho=\rho_{0}\left(1+\frac{x^{2}}{\Lambda^{2}}\right)^{-\frac{\Lambda^{2}}{2\sigma_{x}^{2}}}$ (S9)

This is Gaussian when $\Lambda>>\sigma_{x}$and otherwise has a Gaussian core and an exponential tail. If the range of attraction, $\Lambda$, grows more rapidly than linearly with swarm size$\sigma_{x}$then smaller swarms will have heavy-tail density profiles and larger swarms will have Gaussian density profiles, in accordance with observations [van der Vaart, Private Communication].

**S6. Accounting for non-Gaussian velocity statistics**

Kelley and Ouellette [2013] reported that standardized probability density functions of horizontal and vertical velocities have Gaussian-shaped cores and exponential-like tails that grow monotonically with swarm size. This trend is clearer in the speed distributions. Kelley and Ouellette [2013] reported that their small swarms, like the small swarms of Okubo [1986], agree well with Maxwell-Boltzmann statistics (which pertain to ideal gases close to thermodynamic equilibrium); for their larger swarms, however, the speed distributions show long, nearly exponential tails that grow monotonically with swarm size. Kelley and Ouellette [2013] suggested that the deviations from Gaussian and Maxwell-Boltzmann statistics can be attributed to clustering [Supplementary Material 3]; that is, to the non-uniform distribution of the individual insects within the swarm. Here it is shown how the observations of Kelley and Ouellette [2013] can be attributed to changes in the velocity statistics at the swarm edge. Such changes in velocities are predicted by the new stochastic model, Eqn. 7, (Fig. 1) and are expected on biological grounds because individual insects are free to join or leave swarms and because the velocities of solitary insects (new joiners) are generally higher than the velocities of insects within swarms [Puckett and Ouellette, 2014].

When velocity variances, $\sigma_{u}^{2}$, are position-dependent, the unconditional probability density function for velocity is determined by

$P\left(u\right)=\frac{1}{2\pi\sigma_{x}}\int_{-\infty}^{\infty} \frac{1}{\sigma_{u}}\exp\left(-\frac{u^{2}}{2\sigma_{u}^{2}}\right)\exp\left(-\frac{x^{2}}{2\sigma_{x}^{2}}\right)dx$ (S10)

if velocities are locally Gaussian and if, consistent with observations [Okubo 1986, Kelley and Ouellette 2013], the swarm has a Gaussian density profile. It is readily shown using the saddle point approximation that if $\sigma_{u}^{2}$increases monotonically with distance from the swarm centre then$P\left(u\right)$ will have Gaussian-shaped cores and long, nearly exponential tails that grow monotonically with increasing swarm size (Fig. S3a) in accordance with observations [Kelley and Ouellette 2013]. Furtherover, in accordance with observations [Kelley and Ouellette 2013], standardized speed distributions have Maxwellian cores and long, nearly exponential tails that grow monotonically with swarm size (Fig. S3b). Predictions are shown for the illustrative case where the velocity variance $\sigma_{u}^{2}=1$a.u. when $\left|x\right|<2\sigma_{x}$ and $\sigma_{u}^{2}=1+\left(\left|x\right|-2\sigma_{x}\right)$ when$\left|x\right|\geq2\sigma_{x}$ (Fig. S3c); which is broadly consistent with the new stochastic model, Eqn. 7 (Fig. 1) and which from a biological perspective allows for velocity matching across the outskirts of the swarm, i.e., between individuals inside and outside of the swarm. This analysis is not realistic in detail but is illustrative of a general principle. Conversely, if within-swarm velocities are greater than external velocities then the tails of $P\left(u\right)$ are predicted to narrow with increasing swarm size, contrary to experiment [Kelley and Ouellette 2013].

**
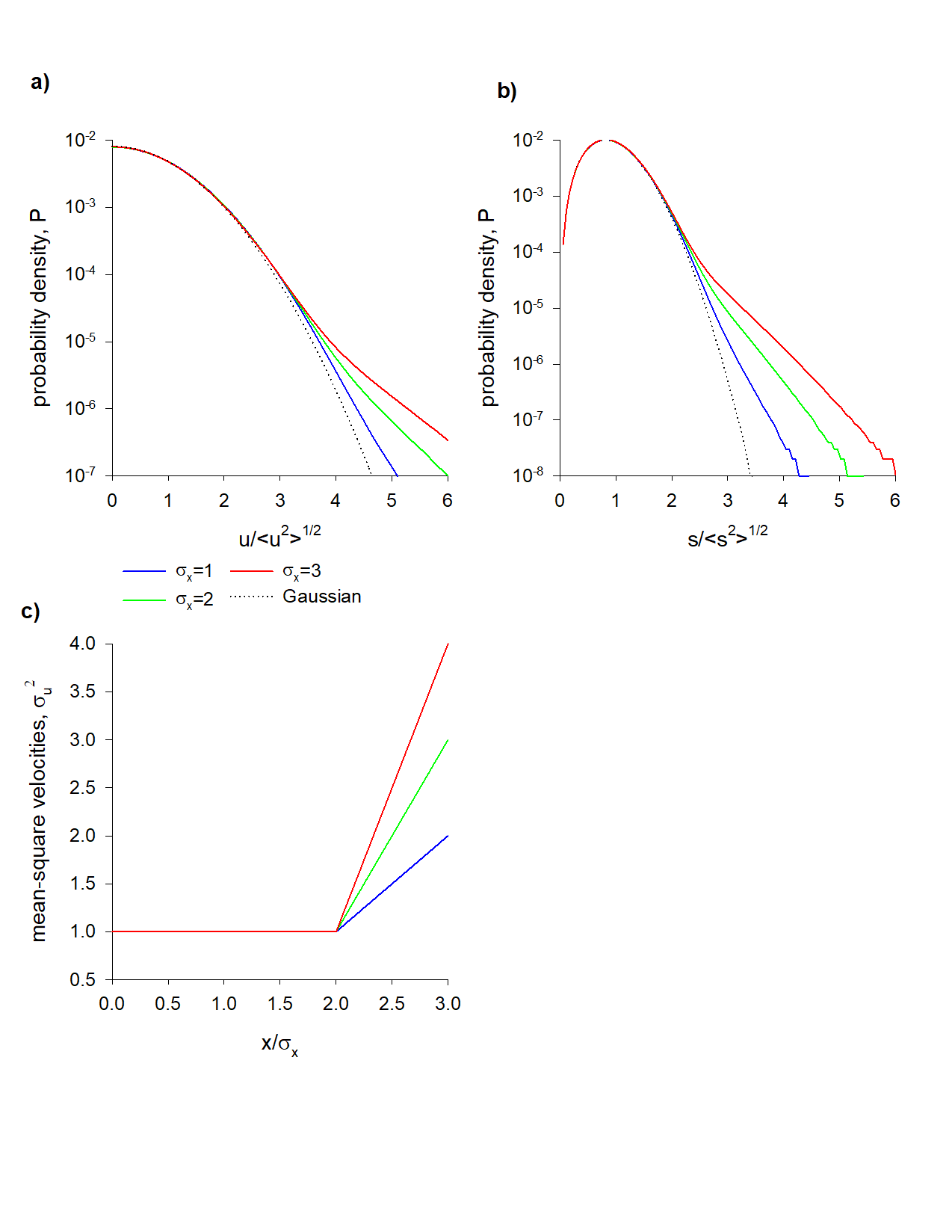
**

**Figure S3a,b) Predicted forms of the position-averaged velocity and speed distributions for swarms with spatially-dependent mean-square velocities displayed in c).**

**S7. The mean surface pressure on an insect swarm**

The scalar virial theorem states that for a self-gravitating system in static equilibrium *2T+W+S=0* where *T* is the kinetic energy, *W* is the potential energy and *S* is the surface pressure. Gorbonos et al. [2016] found that laboratory swarms of the midge *Chironomus riparius* have S<0, indicating that the swarms are effectively experiencing stabilizing inward pressures on their outer surfaces. Gorbonos et al. [2016] reported that “such external stabilizing pressures are commonly found in astrophysical stellar systems, such as globular clusters.” Here I show how that the observed flux of individuals into and out of a swarm will create a flux of momentum that acts as a surface pressure term in *2T+W+S=0* [Kelley and Ouellette 2012, Ni and Ouellette 2016, Sinhuber et al. 2019]. Fluxes of individuals into and out of swarms are, in fact, an inherent feature of the new model because swarms are predicted to be surrounded by low-density clouds of individuals that undergo diffusion [See text relating to Eqn. 1].

In the analogy with self-gravitating systems [Okubo 1986], the predicted contributions to the swarm binding from the surrounding insects correspond to a ‘dark matter halo’; structures that extended well beyond the edges of stellar systems and those existence can be inferred through their effects on the motions of stars and gas within those systems.

Fluxes of individuals into and out of the swarm can be expected to momentarily shift the centre-of-mass of the swarm and cause it to expand and contract. Centre-of-mass movements have, in fact, been observed in the laboratory but these movements may be due, in part, to the limited number of individuals within the swarms and to other processes [Reynolds and Ouellette 2016]. A simple one-dimensional stochastic model that accounts for centre-of-mass movements can be formulated using the approach of Reynolds and Ouellette [2016]. This model is given by

$\begin{matrix} du=\frac{d\overline{u}}{dt}dt-\frac{\left(u-\overline{u}\right)}{T'}dt-\frac{\sigma_{u}^{2}}{\sigma_{x}^{2}}\left(x-\bar{x}\right)dt+\sqrt{\frac{2\sigma_{u}^{2}}{T'}}dW \\ dx=udt \end{matrix}$ (S11)

where *x* and *u* are the position and velocity of the insect at time *t*, $T'$is a velocity autocorrelation time and $dW\left(t\right)$ is an incremental Wiener process with correlation property$\overline{dW\left(t\right)dW\left(t+\tau\right)}=\delta\left(\tau\right)dt$. Positions are Gaussian distributed with mean $\overline{x}$ and variance $\sigma_{x}^{2}$and velocities are Gaussian distributed with mean $\overline{u}=\frac{d\overline{x}}{dt}$and variance $\sigma_{u}^{2}$. When centre-of-mass motions vanish, $\frac{d\overline{x}}{dt}=0$, Eqn. S10 reduces the classic model of Okubo [1986].

The kinetic and potential energies are given by

$\begin{matrix} T=\frac{\sigma_{u}^{2}}{2}+\frac{1}{2}\left(\frac{d\overline{x}}{dt}\right)^{2} \\ W=\left\langle xF\right\rangle=-\sigma_{u}^{2} \end{matrix}$ (S12)

It follows that the surface pressure term is given by

$S=-\left(\frac{d\overline{x}}{dt}\right)^{2}$ (S13)

and so is negative, in accordance with observations [Gorbonos et al. 2016]. It can be shown that this result, i.e., that *S=0* for stationary swarms and that *S<0* for time-dependent swarms, holds true generally, irrespective of the form of the swarm density profile and irrespective of the velocity statistics. Changes in swarm size due to fluxes of individuals into and out of the swarm can be modelled in a similar way. In this case, the mean velocity $\overline{u}=\frac{x}{\sigma_{x}}\frac{d\sigma_{x}}{dt}$depends on position and consequently mean speed profiles,

$$\left\langle\left|u\right|\right\rangle=\sqrt{\frac{2}{\pi}}\sigma_{u}\exp\left(-\frac{\bar{u}^{2}}{2\sigma_{u}^{2}}\right)+\bar{u} erf\left(\frac{\bar{u}}{\sqrt{2}\sigma_{u}}\right),$$

are not predicted to be flat in the cores of swarms contrary to observations (speeds vary by a factor of about 1.4 when model parameters are chosen to match experimental estimates of kinetic and potential energies, and surface pressures). This suggests that the dominate contribution to the surface pressure term comes from centre-of-mass movements.

In principle surface confining pressures can be produced by ‘stochastic’ boundaries beyond which outwardly-moving individuals sporadically turn around to commence flying towards the centre of the swarm also result in (results not shown). Such behaviour is biologically plausible because “the distinct and stable properties of the swarm core and outer region may provide a mechanism for the regulation of the swarm edge: an individual may be able to recognise that it has crossed the phase boundary, and that it is therefore time to turn around to remain in the swarm” [Sinhuber and Ouellette 2017].There is, however, no evidence for this in the datasets of Sinhuber et al. [2019]. The likelihood of turning back towards the swarm centre does not vary with distance from the swarm centre when outside of the swarm cores. Hard boundaries at which density profiles fall abruptly to zero also result in confining surface pressures (analysis not shown) but such boundaries seem biologically implausible except perhaps at ground level. The imposition of a single hard boundary at ground-level is incompatible with observations which indicate an isotropic origin of surface pressure [Gorbonos et al. 2016].

**S8. The most tightly-bound wild swarms are predicted to be analogous to black holes**

Okubo [1986] proposed that insect swarms are analogous to self-gravitating systems. Reynolds [2018] subsequently posited a more nuanced proposal: namely that laboratory swarms are analogous to globular clusters, as claimed by Gorbonos et al. [2016], whereas wild swarms are analogous to stars and giant gaseous planets. The distinction arises because the presence of a fluctuating environment drives the formation of transient, local order (synchronized subgroups) and because this local order pushes the swarm as a whole into a new state that is robust to environmental perturbations. The theory of Reynolds [2018] predicts that the aerial density profiles of wild swarms are accurately characterized by q-Gaussians where 2-q is the number of individuals in a synchronized group. Subgroups typically consist of a pair of individuals but subgroups containing many more individuals have been observed, albeit fleetingly [Shishika et al. 2014]. q-Gaussians, also known as polytropic distributions, constitute the simplest, physical plausible models for self-gravitating systems. Gaussians, i.e., q-Gaussians with $q\to1$, correspond to collisionless systems of stars like globular clusters. q-Gaussians with q<1 provide models for stars and giant gaseous planets. Reynolds [2018b] found support for q-Gaussians with q<1 in telemetry data for swarming mosquitoes [Shishika et al. 2014].

Here the theory of Reynolds [2018] is shown to predict that the entropies of the most tightly bound wild swarms are approximately proportional to their surface areas and so in congruence with the entropies of black holes [Hawking 1975]. The analogy between insect swarms and self-gravitating systems is thereby pushed to its’ most extreme limit.

According to the theory of Reynolds [2018] the aerial density profiles of the most tightly bound wild swarms are characterised by q-Gaussians with q’s large and negative, i.e., by

(S14)

where *x* is the distance from the swarm centre and $\sigma_{x}$is a measure of the size of the swarm. In contrast with Gaussian distributions which have long thin tails, the q-Gaussians vanish at the outer surface of the swarm, thereby creating a ‘horizon’: individuals can enter but not exit from the swarm (without first decoupling their dynamics from the swarm dynamics).

If velocities, *u*, are Gaussian distributed and independent of position, as they are to good approximation in laboratory swarms [Kelley and Ouellette 2013], then the Shannon entropy at position, *x*,

$\begin{matrix} S\left(x\right)=-\int_{\infty}^{\infty} P_{x}\left(x\right)P_{u}\left(u\right)\log\left[P_{x}\left(x\right)P_{u}\left(u\right)\right]du \\ =-P_{x}\left(x\right)\log P_{x}\left(x\right)+P_{x}\left(x\right)\log\left[\sqrt{2\pi}e\sigma_{u}\right] \\ =-P_{x}\left(x\right)\log\left[1-\frac{x^{2}}{3\sigma_{x}^{2}}\right]^{-\frac{1}{q}} \end{matrix}$ (S15)

when $\log\left[2\sqrt{3}\sigma_{x}\right]=-\log\left[\sqrt{2\pi e}\sigma_{u}\right]$, i.e., when the constraint $\sigma_{x}\sigma_{u}=\frac{1}{2\sqrt{6\pi e}}$ applies. In this case,

$S\left(x\right)\approx\frac{1}{2\sqrt{3}\sigma_{x}q}\log\left[1-\frac{x^{2}}{3\sigma_{x}^{2}}\right]$ (S16)

The same result, albeit with a different constraint constant, is obtained with other choices for the velocity distribution. Integration of Eqn. S16 over *x* reveals that the dominant contribution to the total entropy (over 50% of the total) comes from a narrow shell of the swarm, the outer shell where (Fig. S4a), and that this contribution does not depend on the size of the swarm, $\sigma_{x}$. It follows that the total entropy is approximately proportional to the area of the swarm (Fig. S4b). This contrasts with the total entropies of less-strongly-bound swarms that are proportional to their volumes because there are no dominant narrow shells.


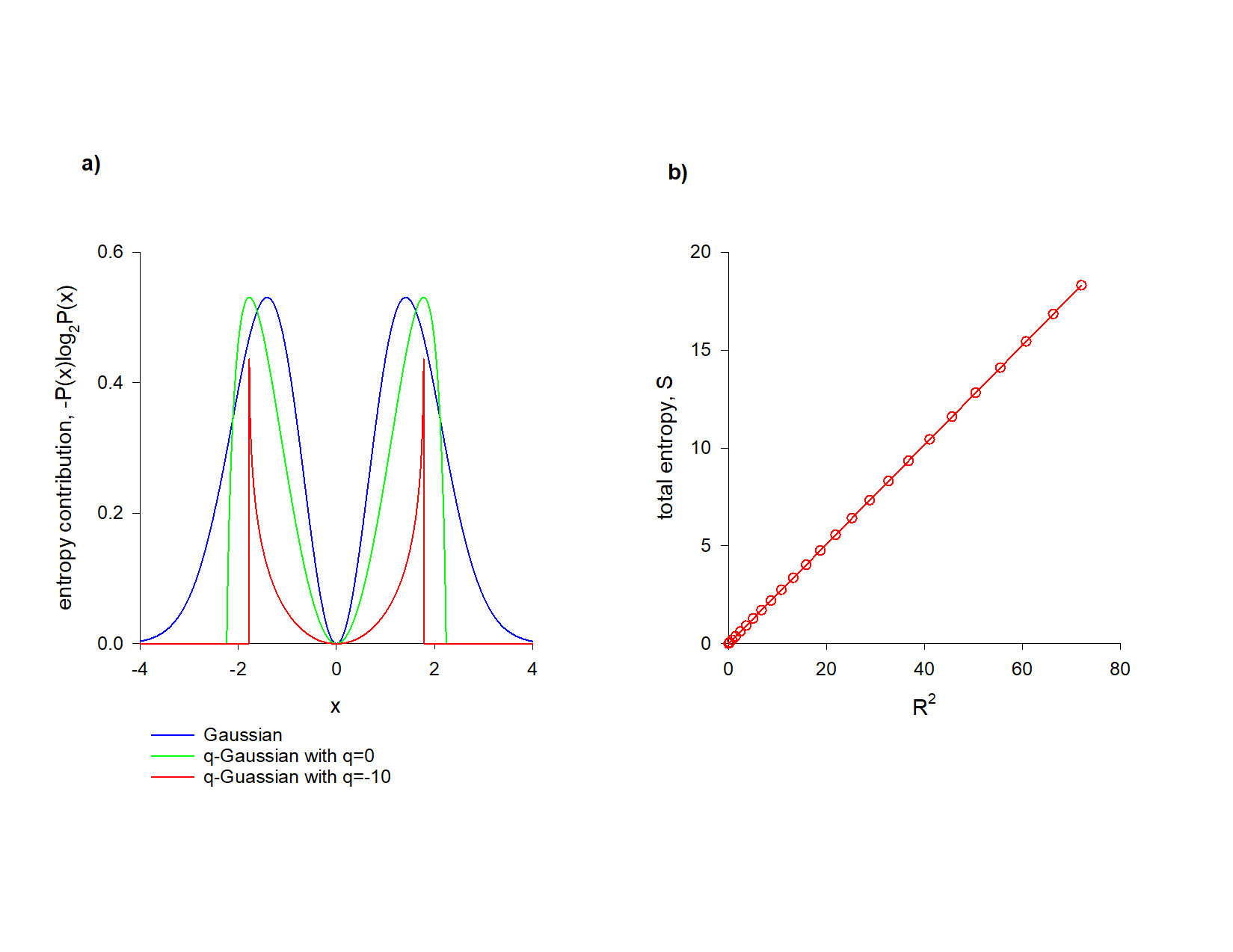


**Figure S4) Entropies of the most tightly-bound wild swarms are predicted to be proportional to their surface areas and are therefore analogous to black holes. a)** The dominant contribution to the entropies (in bits) of strongly-bound swarms (characterized by q-Gaussian density profiles with *q* large and negative [Reynolds 2018b]) are predicted to come from their outer shells. Shown for comparison are the more distributed-contributions to the total entropies of less-strongly-bound swarms **b)** The total entropies (in bits) of strongly-bound swarms (with *q=-10*) is proportional to their surface areas, $4\pi R^{2}$where $R=\sqrt{3}\sigma_{x}$ (o). The line is added to guide the eye. Entropies were calculated numerically for 3-dimensional spherical-symmetric swarms.

**S9. Supernova-like instabilities**

Some external sounds can trigger the explosive dispersal of laboratory swarms of the midge *Chironomus riparius* [Sinhuber and van der Vaart, Private Communication]. Here it is shown that this instability may be attributed to a proliferation of pairwise interactions which might be expected if the males are falsely altered to the presence of females. Exploding insect swarm may therefore be analogous to supernovas as both kinds of events are caused by the sudden ignition of fusion reactions.  This is just one possible mechanism, awaiting experimental testing. Another possibility is that external noise disrupts (breaks) the coupling between individuals, causing the swarm to break-up.

Midge swarms are composed exclusively of males and are epigamic. It is thought that males locate females within the swarm by listening for their characteristic wingbeat sounds which distinguish them from males. Males are therefore highly sensitive to acoustic stimuli and as consequence swarms can be perturbed by external sounds [Ni et al. 2015]. Midges typically spend about 15% of their time engaged in nearly harmonic oscillatory flights conducted in synchrony with another midge (velocities tend to be antiparallel) [Puckett et al. 2015]. Puckett et al. [2015] suggested that these interactions were used for determining the gender of other individuals. Flying back and forth passed another midge may be a way for an individual to isolate the sound of that midge from the background hum of the rest of the swarm. The fission-fusion model, Eqn. S3, describes the long-time dynamics of swarms where this behaviour proliferates, induced perhaps by an external sound source that is mistaken for the presence of females. The 3-dimensional solution to Eqn. S3

$N\left(r,t\right)=\left(Dt\right)^{-6}\left[1+\left(Dt\right)^{-4}r^{2}\right]^{-2}$ (S17)

shows that the proliferation of bound anticorrelated (non-diffusive) pairs triggers very rapid expansion of the swarm. The width,$\Delta$, of the swarm is predicted to grow super-ballistically over time according to $\Delta=2\sqrt{3}\left(Dt\right)^{4}$, i.e., the expansion of the swarm is accelerating over time. This expansion is predicted to cease when dilution precludes pairwise interactions.

By way of contrast, proliferation of correlated (diffusive) subgroups of individuals is predicted to result in tightly-bound swarms that are analogous to black holes [Supplementary Material S9]. These findings are consistent with the analysis of Reynolds [2018b] which predicts that the presence of correlations intensifies the resultant attraction to the swarm centre, whilst the presence of anticorrelates weakens it.

**S10. Midge swarms have anti-thermodynamic properties in common with self-gravitating systems**

Sinhuber et al. [2019] reported on the response of insect swarms in a laboratory experiment where they had full control over external perturbations. They considered the effect of controlled variable light exposure on the swarming behaviour. They found that individuals respond to light changes by speeding up during brighter conditions and that the swarm responds to the perturbations by compressing and simultaneously increasing the attraction of individual midges to the swarm’s centre of mass. Sinhuber et al. [2019] obtained a quantitative physical understanding of this consistent swarm-level response by making an analogy to thermodynamics, with the state effectively moving along an isotherm in the thermodynamic phase plane. This was done by relating changes in density to changes in microscopic pressure per unit mass defined as

$\Pi=\frac{1}{3NV}\sum_{i=1}^{N} u_{i}^{2}+A_{i}.x_{i}$ (S18)

where$x_{i}, u_{i}$and $A_{i}$ positions (relative to the centre of mass), velocities and accelerations of individual insects. This definition captures the work done by insects moving in the effective potential well of the swarm. The observed isothermal swarm-level response is predicted by the model of Reynolds et al. [2017] following a compression (a startle response to a change in light level) (Fig. S5a-c). By way of contrast when the perturbation consists of both a compression and a loss of a few of individuals from the swarm, the swarm-level response is predicted to be non-adiabatic (Fig. S5d) and ‘anti-thermodynamic’ since pressure and so temperature increases when heat (energy) is carried away from the swarm by displaced individuals (a process akin to evaporation). Self-gravitating systems are also anti-thermodynamic, i.e., have negative heat capacities [Eddington 1920]. This similitude may help to an establish a "thermodynamic" description of swarms, as advocated by Ouellette [2017]. In accordance with observations [van der Vaart, Private Communication] the model of Reynolds et al. [2017] predicts that average pressures$\left\langle\Pi\right\rangle\propto N^{-3/2}$.


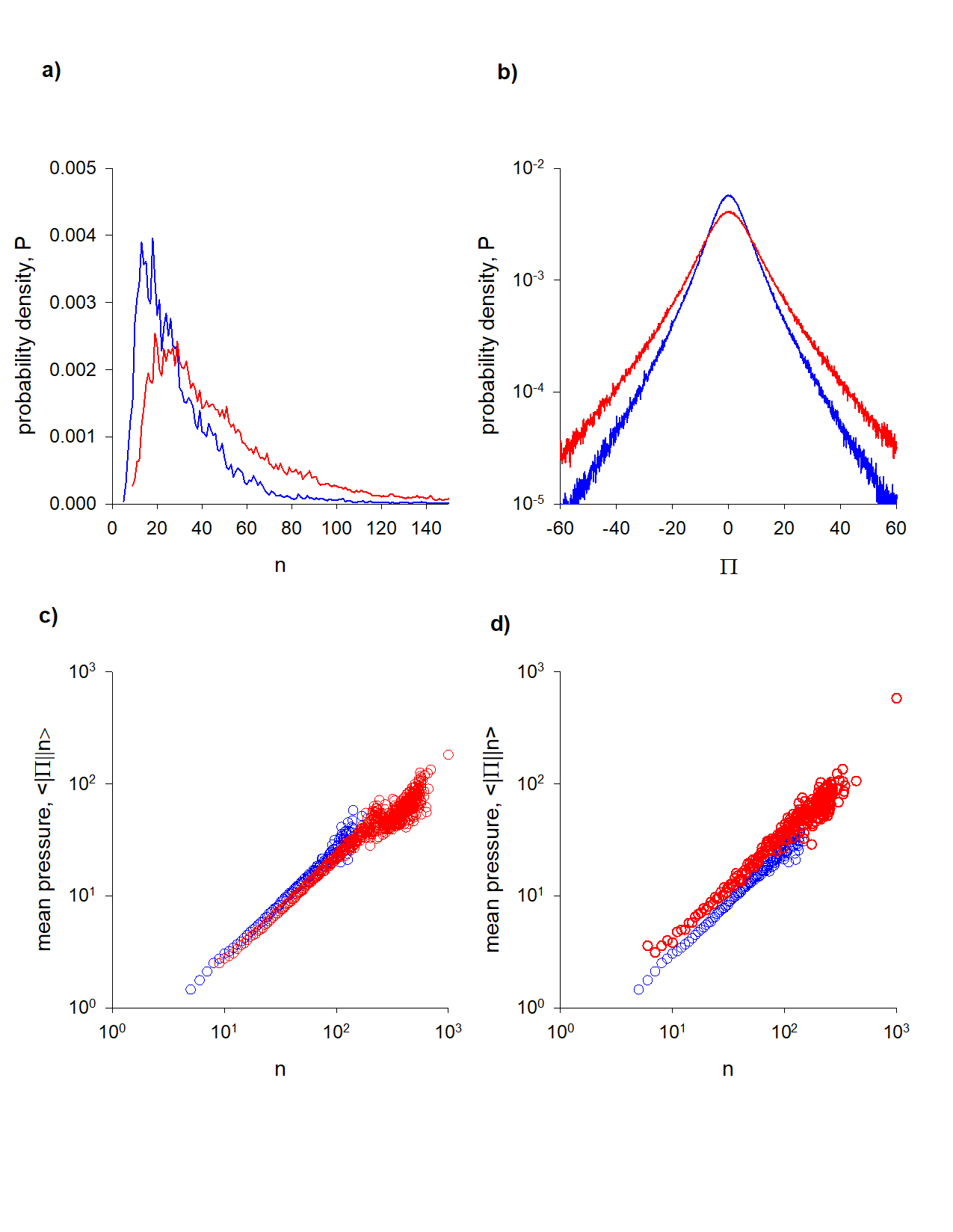


**Figure S5) Predicted swarm densities and pressures before (blue) and after (red) a compression. a) Probability density functions for swarm densities and b) pressures. c) Pressure as a function of density showing that compression moves the state of the swarm along an isotherm. d) A non-adiabatic response is predicted to arise when compression is accompanied by the loss of a few (~30%) individuals from the swarm.**

Predictions are shown for the model of Reynolds et al. [2017] with $\sigma_{u}=1, \sigma_{r}=1$ and *T=1* a.u. N=38 individuals were tracked simultaneously. Compressions reduced the swarm volumes by 25%. Pressures and densities were monitored over 1 a.u. of time after compression.

**S11. A putative origin of inertia**

When driven by an oscillatory visual cue laboratory swarms of the midge *Chironomus riparius* respond as if they are viscoelastic [van der Vaart et al. 2019]. The behavioral response of midges to the motion of conspecifics endows the swarms with an effective inertia. The emergence of effective inertia is predicted by the stochastic model of Reynolds et al. [2017]. Nonetheless, the origins of this effective inertia have until now remained elusive. Here I show how this effective inertia can arise if the interactions of individuals with the rest of the swarm are like the interactions of electric charges and currents with an electromagnetic field, i.e., if the interactions are ‘gravito-electromagnetic’ rather than gravitational *per se*. The approach draws heavily on the analysis of Sciama [1953] who showed that local inertial reaction forces could, through the influence of gravity, be determined by the motion of distant objects. This is a form of Mach’s Principle.

The strength of the “gravito-electric” field, the gravitational counterpart of the electric field, is given by

(S19)

where

and

$\mathbf{A}\left(\mathbf{x}\right)=\frac{G}{c}\int\rho\left(\mathbf{x}'\right)\mathbf{v}\left(\mathbf{x}'\right)/\left|\mathbf{x}-\mathbf{x}'\right|d^{3}x'$ (S20)

are the scalar and vector potentials, *G* is a constant (setting the strength of the interactions),$\rho$is the mass charge density, **v** is velocity (of an individual at *x’* relative to an individual at *x*) and *c* is the speed of propagation of the gravito-electric field. The gravito-magnetic field $\mathbf{B}=\nabla\wedge\mathbf{A}$does not act strongly because midges do not circulate (orbit) in a preferred direction [van der Vaart and Sinhuber, Private Communication] and so the curl of the vector potential vanishes (on average).

From the perspective of any one individual, moving with velocity **v** with respect to the swarm’s center of mass, every part of the swarm appears, albeit approximately, to be moving rigidly with velocity **-v**. Consequently,

(S21)

The gravito-electric forces operating within swarms are “adaptive” rather than Newtonian, being relatively weak in the cores of the swarms where densities are relatively high and being relatively high in the outskirts of swarms where densities are relatively low [Gorbonos et al. 2016]. Adaptation is crucial for reconciling the observed nearly Gaussian density profiles with resultant centrally-attractive forces that are observed to nearly increase linearly with increasing distances from the swarm centers [Okubo 1986, Kelley and Ouellette 2013, Gorbonos et al. 2016]. For such a linear resultant force to arise with Newtonian-like gravitational interactions, individuals would have to be uniformly rather than with Gaussian distributed throughout the swarm. Nonetheless, at the mean-field level of description adopted here, adaptive gravity in swarms with Gaussian density profiles is equivalent to Newtonian gravity in swarms with uniform density profiles; as both scenarios give rise to the same resultant forces, i.e., to the same scalar potential . Gauss’s law for gravity dictates that for a uniformly-dense, spherically-symmetric swarm of radius *R* subject to Newtonian gravito-electric forces

(S22)

It follows that an individual located at a distance *r* from the swarm center experiences an inward gravito-electric force given by

$E=-\frac{4}{3}\pi G\rho r\left[1-\left(\frac{v}{c}\right)^{2}\right]+\frac{2}{3c^{2}}\pi G\rho\left[r^{2}-3R^{2}\right]\frac{dv}{dt} \text{for} r\leq R$ (S23)

The first term on the right-hand side of Eqn. S23 is the resultant attraction to the swarm center which in accordance with observations increases linearly with distance from the center [Okubo 1986, Kelley and Ouellette 2013] and is speed dependent. The speed dependency is consistent with that predicted by the stochastic model of Reynolds et al. [2017] (Fig. S6). This is different in form from the observed, much-stronger dependency that is predicted by models based on exponential rather than Gaussian velocity statistics [Reynolds et al. 2017]. Exponential velocity statistics have been attributed to clustering of individuals [Kelley and Ouellette 2012] and to edge effects [Supplementary Material S6]. When present these effects could mask/swamp the gravito-electric contributions to the speed dependency of resultant accelerations. The second term describes a gravito-electric force (an inertial reaction force) produced by the swarm that **opposes** the forces (intrinsic and/or external) acting on an individual whenever it accelerates. In this model, an individual’s inertia is therefore determined by the motion of the rest of the swarm and is a purely collective phenomenon being a biological realization of Sciama’s [1953] formulation of Mach’s Principle. In this picture, distant individuals in the swarm effectively generate a vector potential field throughout the swarm that acts on other individuals whenever intrinsic or external forces cause them to accelerate. In other words, when a focal individual accelerates, gravitational-like disturbances are propagated to all the other individuals in the swarm. These perturbed individuals set up currents that cause disturbances to propagate back to the focal individual to produce an inertial reaction force.

The results of numerical simulations indicate that for relatively large swarms with $\sigma_{x}\geq\sigma_{u}T$, signal speeds are typically about twice the insect flight speeds as might be expected if (as argued in the main text) gravity is an emergent property of pairwise interactions between individuals. In this case, inertial masses at swarm centers, $m_{i}\approx\frac{\sigma_{u}^{2}}{c^{2}}m_{g},$are comparable to gravitational masses, $m_{g}$. Higher signal speeds are, however, predicted for smaller swarms where individual perceptual fields may be continually overlapping so that disturbances can propagate more freely throughout the swarm without the need for significant individual movements (and so not limited by an individual’s speed).


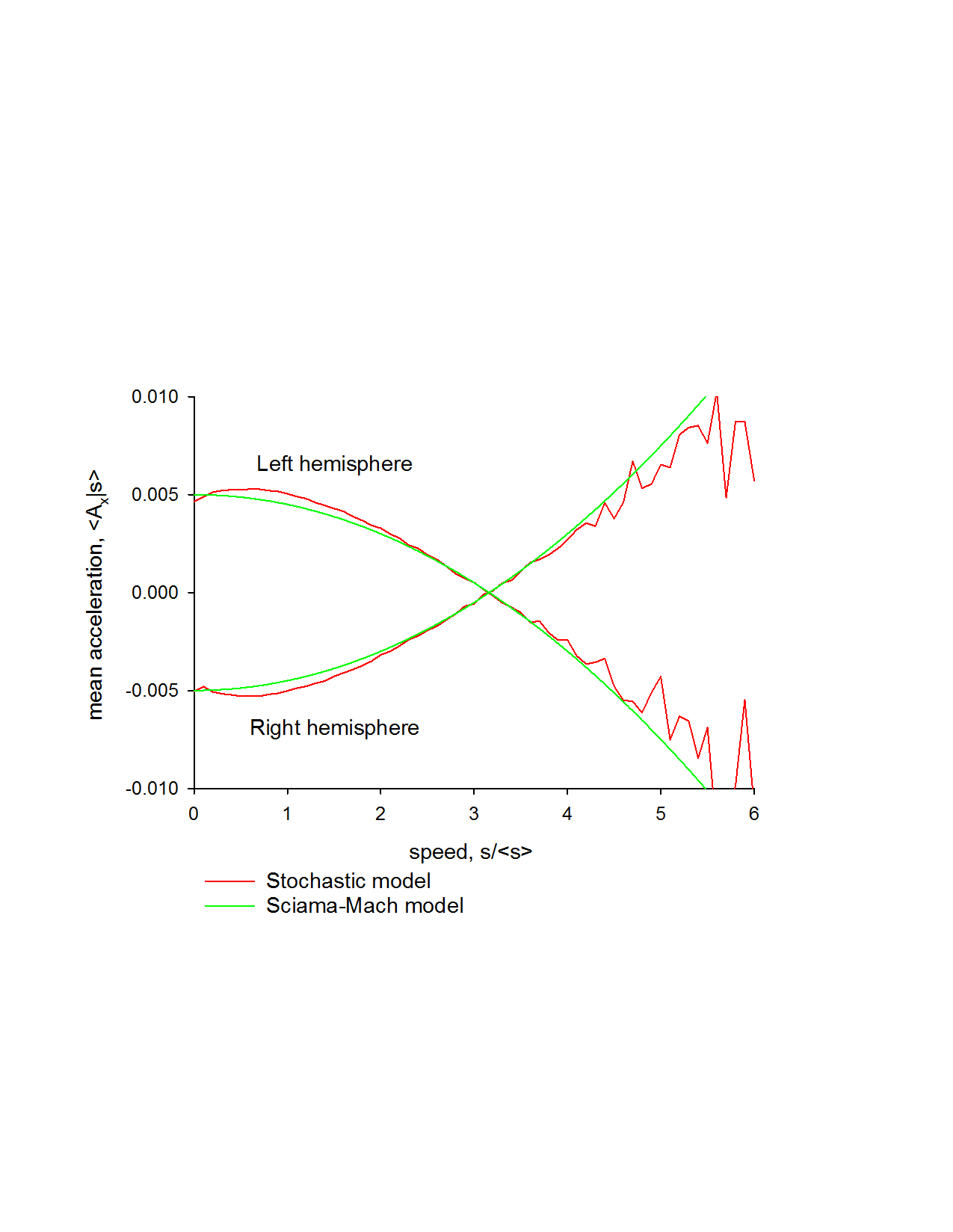


**Figure S6)** **Average value of a single component of the acceleration *A_x_* conditioned on insect flight speed** ***s***, computed using the stochastic model of Reynolds et al. [2017] with $\sigma_{x}=2, \sigma_{u}=1$and $T=1$(red lines) and predicted by the Sciama-Mach model, Eqn. S23 with $c=\sqrt{10}$(green lines).

The switching from inward to outward gravito-electric forces which occurs when individuals outpace gravity is a key prediction and its verification adds further support to the appropriateness of the Sciama-Mach model (Fig. S7). Reynolds et al. [2017] showed that mean accelerations (gravity) increase monotonically with increase speed. This data extended out to about 2.5 times the mean speed. Further analysis suggests that at higher speeds, mean accelerations decrease with increasing speed, perhaps eventually changing sign, in broad agreement with the Sciama-Mach model (Fig. S7).   The outward accelerations need not be construed as being ‘repulsive’ because the results of numerical simulations indicate that they tend to ‘retard’ the motions of fast, inward-moving individuals thereby keeping velocities within accessible (prescribed) realms for self-propelled organisms. That is, individuals experiencing outward accelerations are typically moving towards the centre of the swarm.


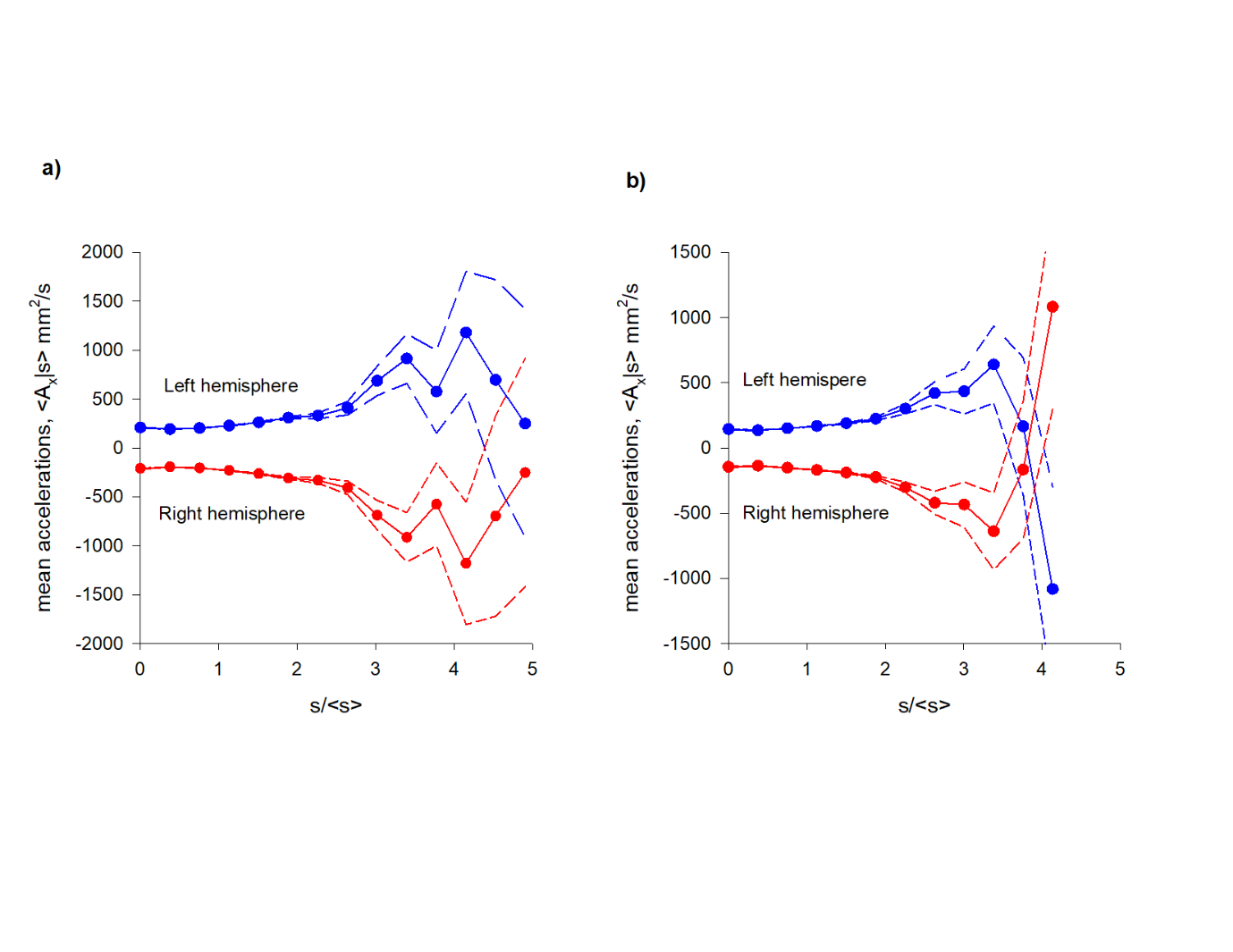


**Figure S7) Average value of a single component of the acceleration *A_x_* conditioned on midge speed** ***s*** computed from experimental data for **a)** small swarms with N<30 individuals and for **b)** large swarms with N>50 (●). The solid-lines are added to guide the eye. The dashed lines represent 95% confidence intervals*.* Data are taken from Sinhuber et al. [2019].

In accordance with the model predictions, Eqns. S23, the velocity-autocorrelation time, a proxy for inertia, decreases with monotonically with increasing distance from the swarm center (Fig. S8a). Inertia is predicted to vanish at locations far from the swarm.


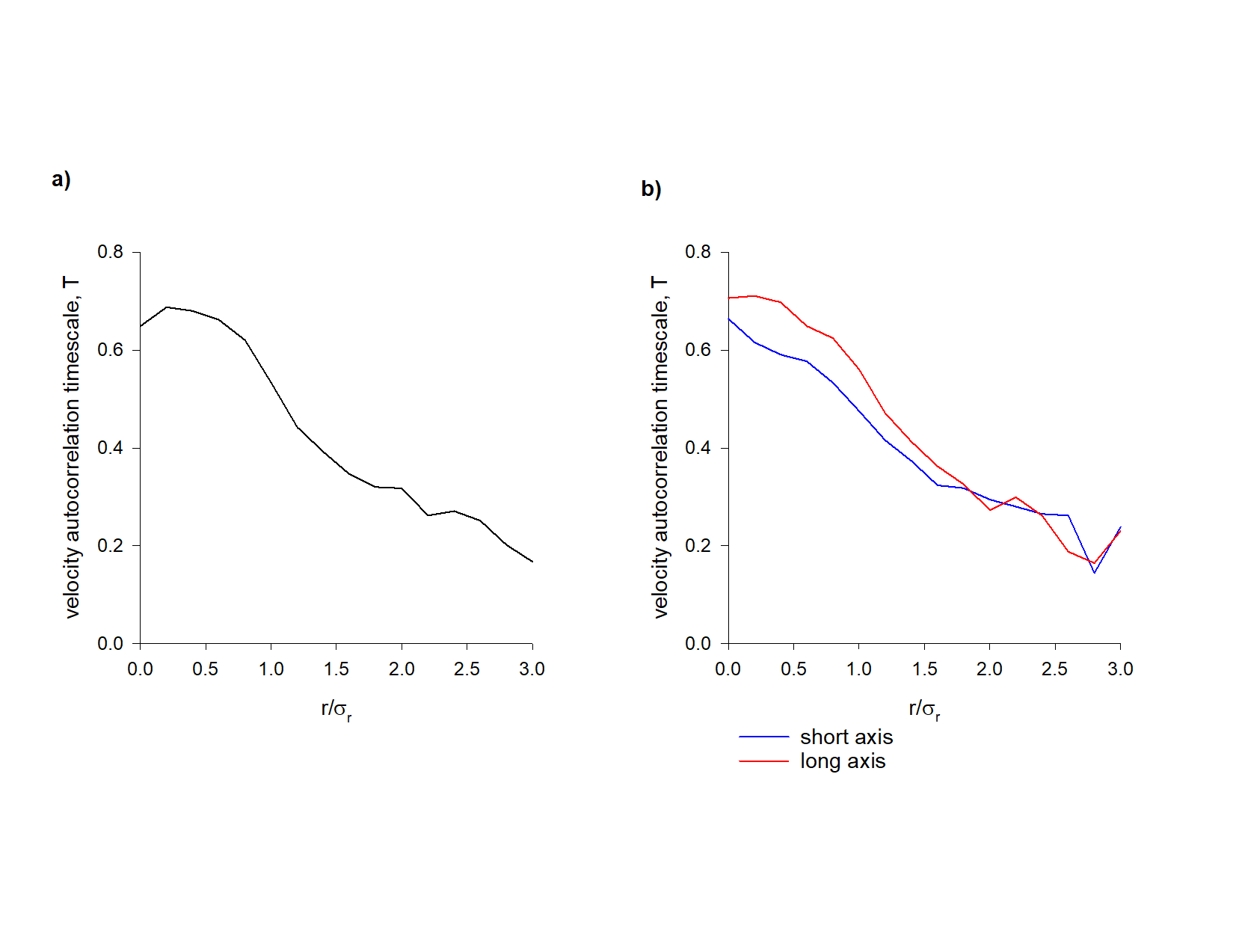


**Figure S8a) In accordance with the Sciama-Mach model, Eqn. S23, the velocity autocorrelation timescale *T*, a proxy for inertia, decreases monotonically with increasing distance from the swarm center, r.** **b) Inertia is anisotropic being larger along the long horizontal axis of the swarm than it is along the short horizontal axis.** The timescale $T=-\Delta t/\ln R\left(\Delta t\right)$where $R\left(\Delta t\right)=\left\langle u\left(t+\Delta t\right)u\left(t\right)\right\rangle/\left\langle u\left(t\right)u\left(t\right)\right\rangle$is the autocorrelation function for one-component for velocity (which to good approximation is approximately exponential $R\left(\Delta t\right)\approx\mathrm{e}^{-\Delta t/T}$ at short times [Okubo 1986]). This is evaluated with $\Delta t=0.1 s$for trajectory-segments starting within annuli with inner and outer radii, *r* and $r+0.2\sigma_{r}$where $\sigma_{r}$is the root-mean-square size of the swarm. Data are taken from Sinhuber et al. [2019]; ensemble averaging over all 17 dusk-time swarms. The average horizontal aspect ratio is 1.1 which to good approximation is the ratio of the velocity autocorrelation times in the cores of the swarms.

Laboratory swarms tend to be weakly axisymmetric [Kelley and Ouellette 2013]. According to Mach’s principle such nonuniform distributions of matter lead to anisotropy of inertia. Velocity autocorrelation timescales (inertia) are, in fact, anisotropic being larger along the longer horizontal axes of the swarms (Fig. S8b). The vertical dimension is not considered here because individuals tend to join swarms by flying above them, thereby extending swarms in the vertical direction [Kelley and Ouellette 2013] and because the Earth’s gravity influences individual movements in that direction.

Gravito-magnetic forces may be present intermittently (but on average they vanish). When present they can be expected to result in rotational trajectories. The presence of such trajectories is consistent with a ‘thermodynamic’ analysis of laboratory swarms which has revealed that individuals have 9 rather than 6 degrees of freedom; 3 translational degrees of freedom, 3 potential and 3 rotational [Sinhuber et al. 2019]. Peaks in the power spectra for rotations of the velocity vector, i.e., **u**^**A**, are also consistent with the intermittent presence of rotational trajectories (Fig. S9a, b).


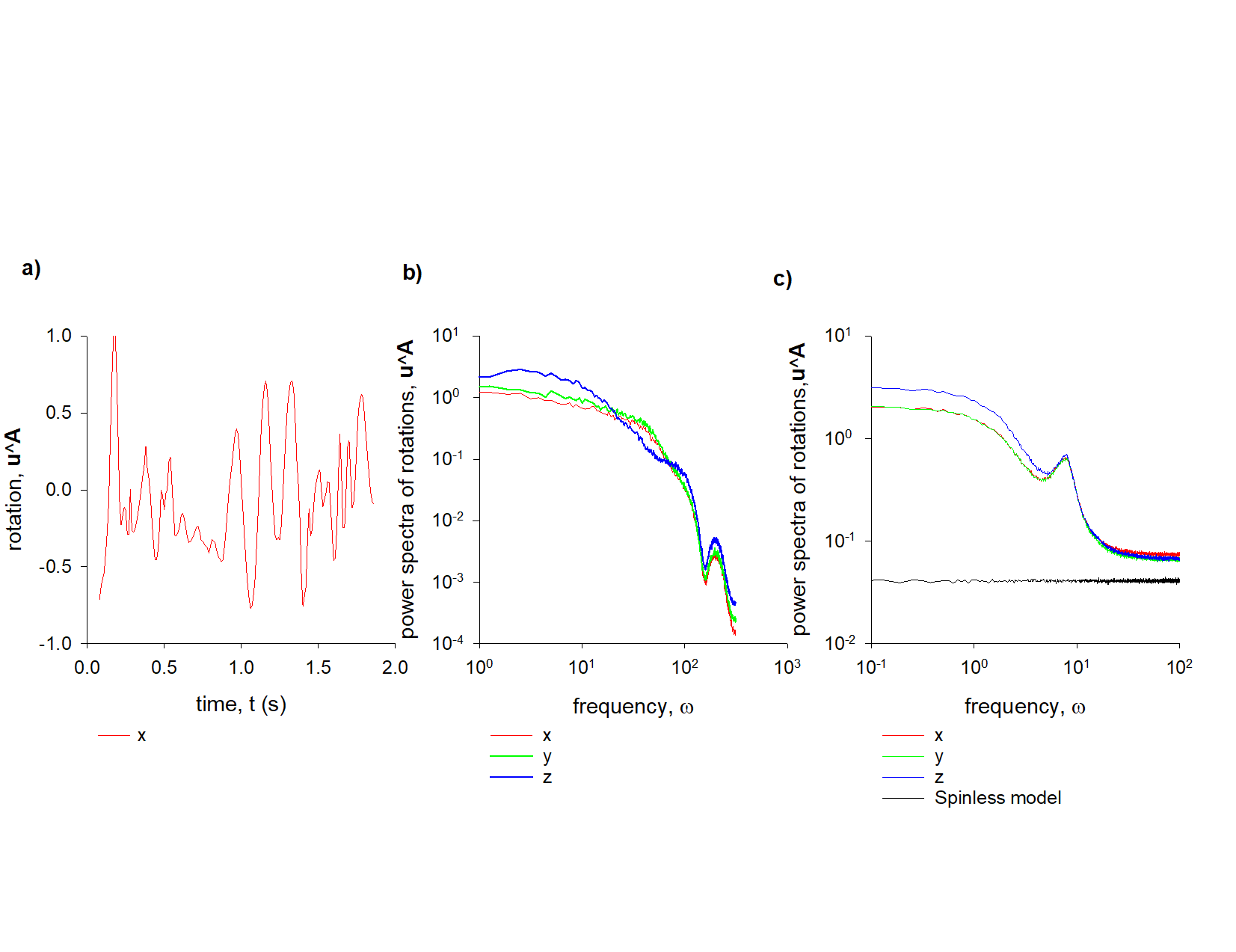


**Figure S9 Evidence for the presence of rotational trajectories in laboratory insect swarms a)** Rotations of the velocity vector of a single midge are indicative of the presence of gravitomagnetic forces with $\left\langle\mathbf{B}\right\rangle=0$as clockwise and anticlockwise rotations are equally prevalent. **b)** Average power spectra of rotations for 17 dusk-time swarms. The high-frequency peaks are indicative of rotations **c).** Power spectra of rotations predicted by the model of Reynolds et al. [2017] (with all model parameters, $\sigma_{R}, \sigma_{u}, T$, set to unity a.u.) with and without rotation-inducing ‘spin’ terms ($\mathbf{\Omega}=\left(4,4,5\right)$). Experimental data are taken from Sinhuber et al. [2019]. Similar predictions are obtained when $\mathbf{\Omega}=\left(\pm4,\pm4,\pm5\right)$.

Further evidence for gravito-magnetic forces comes from comparisons of the observations with predictions from the stochastic models. Stochastic models for the simulation of swarming insect trajectories admit gravitomagnetism. These models take the general form

$\begin{matrix} du_{i}=a_{i}\left(\mathbf{u},\mathbf{x},t\right)dt+bdW_{i} \\ dx_{i}=u_{i}dt \end{matrix}$ (S24)

where **u** and **x** denote the velocity and position of the midge at time *t*, and where $dW_{i}\left(t\right)$is an incremental Wiener process with correlation property $\overline{dW_{i}\left(t\right)dW_{j}\left(t+\tau\right)}=\delta_{ij}\delta\left(\tau\right)dt$. The functional form of deterministic term, $\mathbf{a}\left(\mathbf{u},\mathbf{x},t\right)$, and the magnitude of the driving noise, *b*, can be constrained by the requirement that the simulated trajectories be consistent the observed position-velocity statistics. Mathematically, these consistency conditions are implemented through the Fokker-Planck equation

$\frac{\partial P}{\partial t}+u_{i}\frac{\partial P}{\partial x_{i}}=-\frac{\partial}{\partial u_{i}}\left(a_{i}P\right)+\frac{b^{2}}{2}\frac{\partial^{2}P}{\partial u^{2}}$ (S25)

where$P(u,x,t)$is the joint distribution of velocity and position. In general this equation does not have a unique solution for $a\left(u,x,t\right)$as an arbitrary rotation vector function , satisfying (with ) , can be added to $a_{i}P$. For isotropic, Gaussian velocities (a good approximation for laboratory swarms [Kelly and Ouellette 2013]), the simplest such term corresponds to $a_{i}\left(\mathbf{u},\mathbf{x},t\right)\to a_{i}\left(\mathbf{u},\mathbf{x},t\right)+\varepsilon_{ijk}\Omega_{j}u_{k}$ [Borgas et al. 1997, Sawford 1999]. This additional term endows the simulated trajectories with a ‘handedness’ or chirality about some axes indicated by the direction $\mathbf{\Omega}$which may change over time. This term which is allowed by the mathematics but need not be present therefore reproduces the effects of magnetic fields, i.e., Lorentz forces $\mathbf{F}=\mathbf{v}\wedge\mathbf{B}$where $\mathbf{B}=-\mathbf{\Omega}$. Here this term was added to the stochastic model of Reynolds et al. [2017] for swarming insects making it gravito-electromagnetic rather than gravito-electric. Power spectra of rotations predicted by the gravito-electromagnetic stochastic model resemble observations (Fig.S9c).

The non-linear (quadratic) forms of result in trajectories those handedness is dependent on their direction of travel along some preferred axis [Reynolds 2002]. ‘Corkscrew’ trajectories where, for example, the preferred sense of rotation of trajectories in the 1-2 plane is determined by the direction of motion along the 3 axis, arise when$a_{i}\left(\mathbf{u},\mathbf{x},t\right)\to a_{i}\left(\mathbf{u},\mathbf{x},t\right)+\left\langle A_{i}\left|u\right.\right\rangle$, where

$$\begin{matrix} \left\langle A_{1}\left|u\right.\right\rangle=\Gamma_{1}u_{2}u_{3} \\ \left\langle A_{2}\left|u\right.\right\rangle=\Gamma_{2}u_{1}u_{3} \\ \left\langle A_{3}\left|u\right.\right\rangle=\Gamma_{3}u_{1}u_{2} \end{matrix}$$

with $\Gamma_{1}+\Gamma_{2}+\Gamma_{3}=0$. Corkscrew trajectories were not identified in the empirical datasets of Sinhuber et al. [2019].

**References not listed in main paper**

Eddington, A.S. The internal constitution of the stars. *The Observatory* **43**, 341-358 (1920).

Hawking, S. W. Particle creation by black holes. *Comm. Math. Phys*. **43**, 199–220 (1975).

Sciama, D.W. On the Origin of Inertia. Roy. Astro. Soc. **113** 34–42 (1953).

Sinhuber, M., van der Vaart, K., Feng, Y., Reynolds, A.M. & Ouellette, N.T. An equation of state for insect swarms. (Submitted).
